# Ni^δ+^ Atoms Anchored In Situ on Ultrathin Ni‐Phyllosilicate Nanosheet Ensure High‐Efficient CO_2_ Reduction into CO at Moderate‐Low Temperature

**DOI:** 10.1002/advs.202515872

**Published:** 2025-11-27

**Authors:** Ziluo Ding, Pengfei Li, Qiang Chang, Dongdong Xiao, Xingchen Liu, Wentao Zheng, Hao Yang, Shan He, Fan Wang, Jianguo Wang, Fei Wang

**Affiliations:** ^1^ School of Chemical Science University of Chinese Academy of Sciences Beijing 100190 P. R. China; ^2^ School of Light Industry Science and Engineering Beijing Technology and Business University Beijing 100048 P. R. China; ^3^ Beijing Key Laboratory of Ionic Liquids Clean Process Institute of Process Engineering Chinese Academy of Sciences Beijing 100089 P. R. China; ^4^ School of Chemical Engineering University of Chinese Academy of Sciences Beijing 100190 P. R. China; ^5^ State Key Laboratory of Coal Conversion Institute of Coal Chemistry Chinese Academy of Sciences Taiyuan 030001 P. R. China; ^6^ National Energy Center for Coal to Liquids Synfuels China Co., Ltd Beijing 101400 P. R. China; ^7^ Beijing National Laboratory for Condensed Matter Physics Institute of Physics Chinese Academy of Sciences Beijing 100190 P. R. China

**Keywords:** active microstructures of Ni‐based catalysts, catalytic mechanism, geometric/electronic effects, moderate‐low temperature reverse water‐gas shift, performance optimization

## Abstract

Ni‐based catalysts are considered to be promising candidates for moderate‐low temperature (200−400 °C) reverse water‐gas shift (MLT‐RWGS) as an important CO_2_ reduction pathway. However, their high activation properties for CO inevitably lead to severe methanation at high CO_2_ conversion, creating an activity‐selectivity trade‐off and unsatisfactory CO yields. Here, a novel supported Ni‐based catalyst is deveolped, consisting of abundant Ni^δ+^ atoms anchored in situ on ultrathin Ni‐phyllosilicate nanosheet (a‐Ni^δ+^−PSNS(400), 0< δ ≤1). The a‐Ni^δ+^−PSNS(400) break activity‐selectivity trade‐off and achieve high CO selectivity (92%) toward at a formation rate of 21.0 mmol_CO_ h^−1^ gcat^−1^, outdistancing those of all prevailing Ni‐based catalysts for MLT‐RWGS. Such catalytic performance is attributed to unique geometric/electronic effects of a‐Ni^δ+^−PSNS(400), i.e., exposed monodisperse Ni^δ+^ atoms with low electron density on ultrathin Ni‐phyllosilicate nanosheet. The ultrathin nanosheet enables anchored Ni^δ+^ atoms to fully expose and disperse, boosting atom‐utilization efficiency and atom‐synergistic effects, endowing them with high catalytic activity; while low electron density of Ni^δ+^ atoms extremely weakens their chemical adsorption of CO, preventing further CO hydrogenation into CH_4_, which ensures their high CO selectivity. This work provides new insights into the design of active microstructures of high‐performance Ni‐based catalysts for synchronous high activity‐selectivity.

## Graphical abstract

A novel supported Ni‐based catalyst, consisting of abundant Ni^δ+^ atoms with low electron density anchored in situ on ultrathin Ni‐phyllosilicate nanosheet (a‐Ni^δ+^−PSNS, 0< δ ≤1), achieve simultaneous outstanding CO_2_ conversion and CO selectivity. Their ultrathin nanosheet enables abundant anchored Ni^δ+^ atoms to fully expose and disperse, which can efficiently reduce CO_2_ into CO and weakens the chemical adsorption of CO, preventing CO methanation.

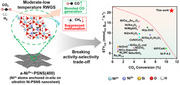

## 1 Introduction

The global climate change caused by carbon dioxide emissions is a common challenge facing humanity.^[^ ^1^, ^2^ ^]^ Converting CO_2_ into value‐added products effectively represents a promising strategy for mitigating CO_2_ emissions.^[^ ^3^ ^]^ The reverse water‐gas shift reaction (RWGS, CO_2_ + H_2_ → CO + H_2_O) through heterogeneous catalysis enables the conversion of CO_2_ into syngas (i.e., CO + H_2_, as an important raw material gas), which can be further converted into liquid hydrocarbon fuel and high value‐added hydrocarbon oxygenates via Fischer−Tropsch synthesis and other syngas processes, e.g., methanol synthesis.^[^ ^4^, ^5^ ^]^ Therefore, the heterogeneous catalytic RWGS is considered as a promising approach for closing the loop of carbon emission and effectively mitigating CO_2_ emissions. The current research mainly focuses on developing high‐performance RWGS catalysts with the goal to improve CO production efficiency.^[^ ^6^, ^7^ ^]^ Notably, owing to their highly potential catalytic properties and relatively low costs, Ni‐based catalysts have been considered as one of the most promising RWGS catalysts.^[^ ^8^, ^9^, ^10^, ^11^ ^]^ To improve the catalytic activity, the Ni‐based catalysts typically require high operating temperatures (exceeding 500 °C), which simultaneously could favor the endothermic RWGS reaction.^[^ ^12^, ^13^, ^14^ ^]^ However, these harsh conditions could cause Ni‐based catalysts sintering and rapid deactivation, as well as a high energetic cost.^[^ ^15^ ^]^ Therefore, numerous studies have strived to develop Ni‐based catalysts with high activity that can operate at moderate‐low reaction temperatures (200−400 °C).^[^ ^9^, ^16^, ^17^ ^]^ Nevertheless, the selectivity of Ni‐based catalysts for moderate‐low temperature RWGS (MLT‐RWGS) reaction is often inevitably limited due to their high activation ability for H_2_ and CO and the resulting severe methanation.^[^ ^8^, ^18^, ^19^ ^]^ Designing Ni‐based catalysts for boosting MLT‐RWGS reaction but avoiding methanation to achieve high activity and high CO selectivity is a crucial task.

Notably, the RWGS reaction and its accompanying methanation possess significant structural sensitivity.^[^ ^8^, ^20^ ^]^ Thus, numerous strategies to intentionally manipulate the microstructure of Ni‐based catalysts, such as increasing metal−support interaction, decreasing particle size, and forming nanoalloy, have been explored to adjust the geometric/electronic effect of active sites, regulating their H_2_ activation and CO adsorption properties, suppressing the methanation and driving the preference for reaction pathways toward RWGS reaction.^[^ ^8^, ^16^, ^21^ ^]^ Typically, preparing ultrasmall Ni particles can reduce the surface step sites that boost C─O bond cleavage; while establishing strong metal−support interaction or nanoalloy phase can create certain superficial or interfacial Ni sites (i.e., Ni^δ+^) with low electron density, weakening the chemisorption and activation of CO and H_2_, thereby suppressing the deep hydrogenation of ^*^CO intermediates involved in methanation and driving the reaction pathway toward the RWGS reaction.^[^ ^16^, ^22^, ^23^ ^]^ However, these strategies reduce largely the active site loading and decrease the density/number of surface available active site, resulting in an obvious decrease in integral activity as CO selectivity increases.^[^ ^13^, ^24^, ^25^ ^]^ Moreover, these strategies not only typically involve complex preparation processes but also give rise to complicated surface/interface structures and the resulting elusive catalytic effect, making it difficult to precisely regulate the microstructure of the catalyst and obstructing optimization of geometric/electronic effect. These, thus, lead to an irreconcilable trade‐off between activity and selectivity of Ni‐based catalysts in MLT‐RWGS, as well as their unsatisfactory CO yields.^[^ ^9^, ^26^, ^27^ ^]^ As a result, developing efficient and controllable strategies that can tailor the geometric/electronic microstructure of Ni‐based catalysts, so as to create abundant exposed active sites with a favorable chemical state, would be valuable for breaking such activity‐selectivity trade‐off and achieving a simultaneous high CO selectivity at high CO_2_ conversion.

**Figure 1:**
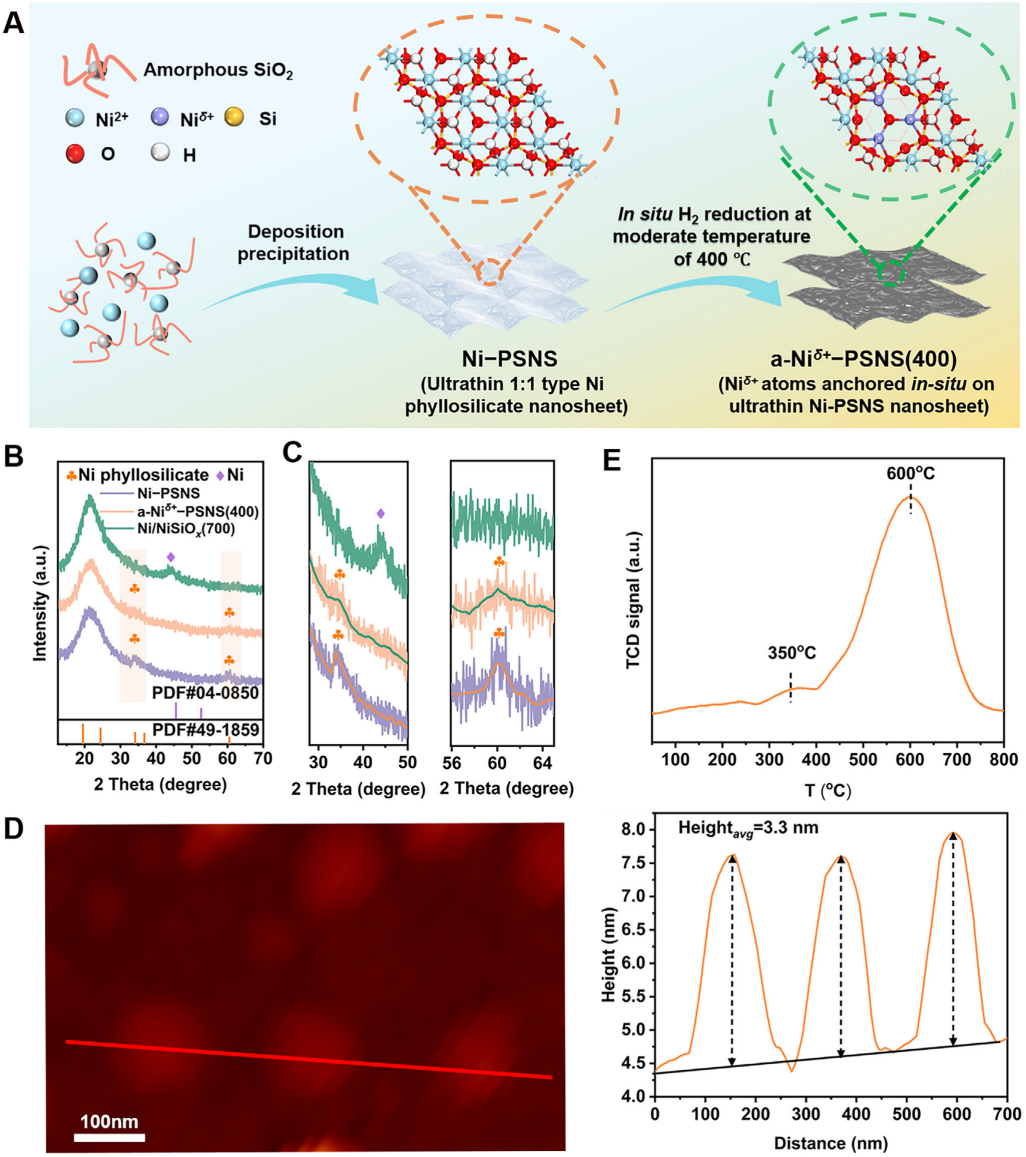
A) Schematic illustration of the preparation of the a‐Ni*^δ+^*−PSNS(400) and their microstructure. B) *Semi‐*in situ XRD patterns of Ni−PSNS, a‐Ni*^δ+^*−PSNS(400), and Ni/NiSiO*_x_* (700). C) *Semi‐*in situ XRD patterns selected from 2θ = 28°−50° and 2θ = 56°−65° for three samples respectively. D) AFM image and corresponding height profile of Ni−PSNS. (E) H_2_‐TPR profiles of Ni−PSNS.

Recently, Ni phyllosilicate has attracted considerable attention as a highly promising precursor for constructing efficient Ni‐based catalysts due to its unique lamellar structure.^[^ ^28^ ^]^ Ni phyllosilicate can be divided into two types according to the ratio of tetrahedral to octahedral layers, i.e., 1:1 type (Ni_3_Si_2_O_5_(OH)_4_) and 2:1 type (Ni_3_Si_4_O_10_(OH)_2_).^[^ ^28^ ^]^ The 1:1 type Ni phyllosilicate possesses a double layered structure composing of one Ni−O(OH) octahedron layer and one Si−O−Si tetrahedron layer; while the 2:1 type Ni phyllosilicate has a sandwich lamellar structure with two Si‐O‐Si tetrahedron layers and one internal Ni‐O(OH) octahedron layer.^[^ ^28^ ^]^ A key structural characteristic of Ni phyllosilicate is that the Ni^2+^ cations are distributed in a uniform manner in the layered structure, enabling it to be used as a precursor for forming supported Ni nanoparticles with small nanoscale and high dispersion and for constructing Ni nanocatalysts with specific surface morphology/structure.^[^ ^29^, ^30^ ^]^ As a result Ni‐based catalysts obtained from Ni phyllosilicate precursor have been widely applied to many important catalytic reaction, such hydrogenation reactions, hydrogenolysis reactions, and methane conversion reactions.^[^ ^31^, ^32^, ^33^ ^]^ In this work, inspired by the unique layered structure of Ni phyllosilicate, abundant Ni*^δ+^* atoms with low electron density anchored in situ on ultrathin Ni‐phyllosilicate nanosheet (denoted as a‐Ni*^δ+^*−PSNS(400), 0< δ ≤1) are skillfully obtained through a convenient two‐step procedure, including construction of ultrathin 1:1 type Ni phyllosilicate nanosheet (Ni−PSNS) precursor (the thickness of ≈3.3 nm) and subsequent in situ reduction at moderate temperature of 400 °C (**Figure** **1**A). Experiments combined with theoretical studies demonstrate that a‐Ni*^δ+^*−PSNS(400) provides abundant Ni*^δ+^* atoms with unique geometric/electronic effects, effectively modulating their H_2_ activation and CO chemisorption properties, and exhibiting highly favorable catalytic behavior for MLT‐RWGS. Their ultrathin nanosheet structure enables abundant anchored Ni*^δ+^* atoms to fully expose and disperse, enhancing atom‐utilization efficiency and atom‐synergistic effects. Accordingly, Ni*^δ+^* atoms can efficiently convert CO_2_ into CO through a redox mechanism, while adjacent Ni*^δ+^* atoms can effectively dissociate H_2_, promoting the redox cycle. Moreover, the low electron density of Ni*^δ+^* atoms extremely weakens their chemical adsorption of CO, preventing further ^*^CO intermediate hydrocracking and subsequent CO methanation, and also accelerating Ni*^δ+^* sites to start a new turnover and MLT‐RWGS catalysis. Meanwhile, Ni*^δ+^* atoms also effectively suppress competitive CO_2_ hydrogenation to CH_4_. Therefore, in sharp contrast to other Ni‐based catalysts, a‐Ni*^δ+^*−PSNS(400) successfully breaks through the irreconcilable activity‐selectivity trade‐off, achieving simultaneous high activity and high CO selectivity for MLT‐RWGS. Correspondingly, the catalytic performance of a‐Ni*^δ+^*−PSNS(400) outdistances all prevailing Ni‐based catalysts for MLT‐RWGS previously reported. This work develops a new strategy in tailoring active sites of Ni‐based catalysts with high activity and desired product selectivity.

## 2 Results and Discussion

### 2.1 Synthesis and Structural Characterization of a‐Ni*^δ+^*−PSNS(400)

**Figure 2:**
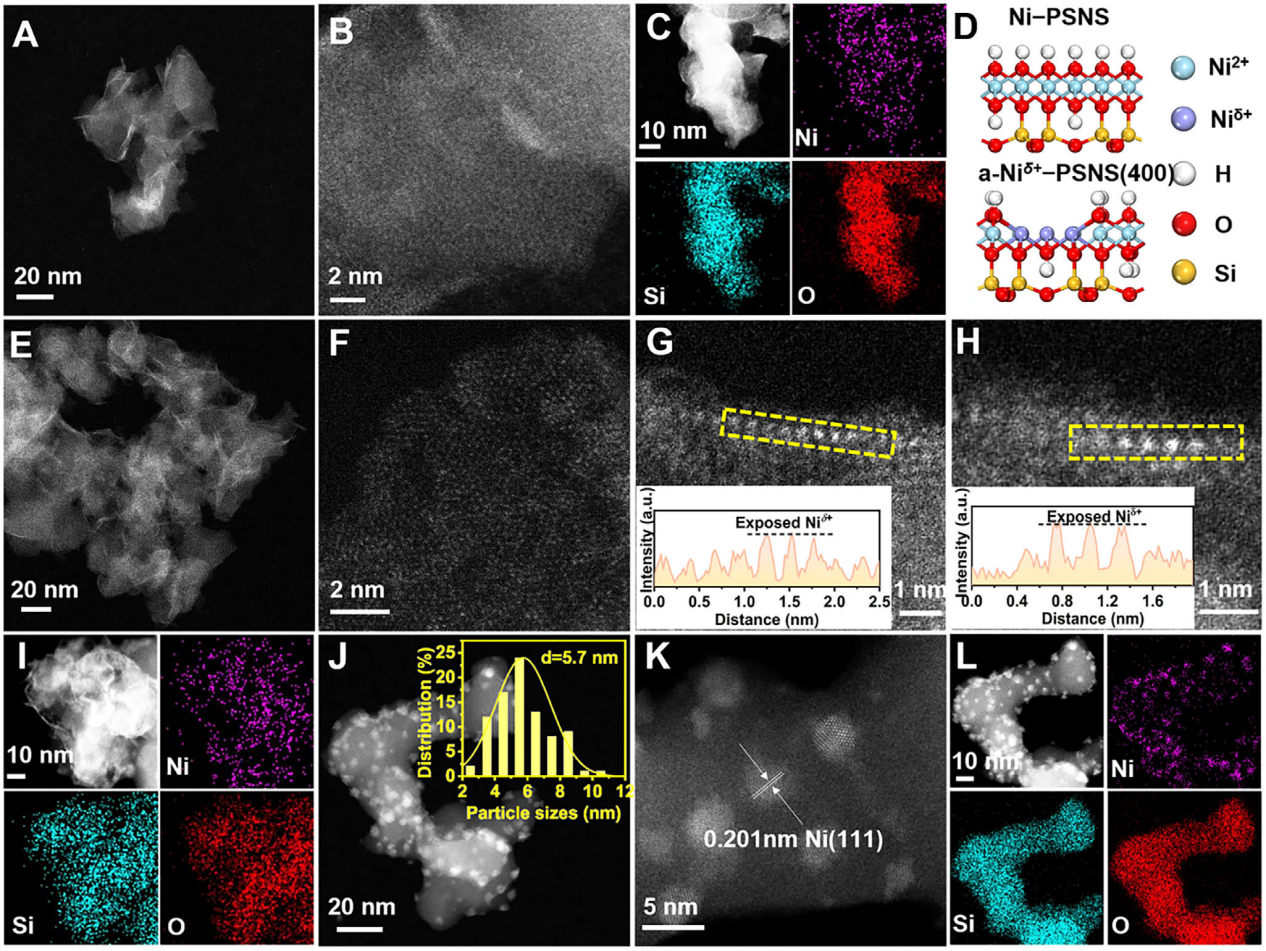
A) STEM image and B) AC‐HAADF‐STEM image of Ni−PSNS. C) EDS elemental mapping images of Ni−PSNS. D) Schematic illustration of the microstructure for Ni−PSNS and a‐Ni*^δ+^*−PSNS(400). E) STEM images and F) AC‐HAADF‐STEM images of a‐Ni*^δ+^*−PSNS(400). G and H) AC‐HAADF‐STEM images of Ni*^δ+^* atoms anchored on two different selected regions of Ni phyllosilicate nanosheet, and the extracted line intensity profiles along the yellow rectangle. I) EDS elemental mapping images of a‐Ni*^δ+^*−PSNS(400). J) STEM image (Inset: histogram of size distribution of supported Ni nanoparticles), K) AC‐HAADF‐STEM image, and L) EDS elemental mapping images of Ni/NiSiO*_x_* (700).

Abundant Ni*^δ+^* atoms with low electron density anchored in situ on ultrathin Ni‐phyllosilicate nanosheet (denoted as a‐Ni*^δ+^*−PSNS(400), 0< δ ≤1) were obtained via a convenient two‐step procedure: preparation of ultrathin 1:1 type Ni phyllosilicate nanosheet (Ni−PSNS) precursor by using homogeneous deposition−precipitation of Ni species on amorphous SiO_2_, followed by a further in situ reduction treatment at moderate temperature of 400 °C (Figure 1A and see Experimental Section in detail). First, during the deposition−precipitation of Ni species onto amorphous SiO_2_, a Ni−PSNS precursor was prepared, which showed a characteristic feature of 1:1 type Ni phyllosilicate structure (PDF#49‐1859, a formula of Ni_3_Si_2_O_5_(OH)_4_), which possessed a double layered structure composing of one Ni−O(OH) octahedron layer and one Si−O−Si tetrahedron layer, as revealed by the *semi‐*in situ X‐ray diffraction pattern (*semi‐*in situ XRD) (Figure 1B,C).^[^ ^34^ ^]^ Atomic force microscopy (AFM) revealed the thickness of Ni−PSNS precursor to be ≈3.3 nm, suggesting their ultrathin 2D nanosheet architecture (Figure 1D). Moreover, aberration‐corrected high‐angle annular dark‐field scanning transmission electron microscopy (AC‐HAADF‐STEM) analyses of Ni−PSNS precursor further confirmed their ultrathin nanosheet morphology (**Figure** **2**A,B,D). Meanwhile, energy dispersive X‐ray spectroscopy (EDS) elemental mapping analysis demonstrated a homogeneous distribution of Ni, Si, and O within nanosheets (Figure 2C). In addition, the reducibility of Ni−PSNS precursor was further investigated by the H_2_ temperature‐programmed reduction (H_2_−TPR) (Figure 1E). H_2_−TPR profile revealed two H_2_ consumption peaks: the small peak located around 350 °C was assigned to the reduction of the outermost hydroxyl species of Ni−O(OH) octahedron layer in Ni−PSNS, which could lead to the exposure of sub‐outer Ni sites and their reduction to Ni*^δ+^* atoms; while the main peak around 600 °C was attributed to the reduction of Ni species with oxidation states in strong interaction with the resulting SiO_2_ phase during in situ reduction process.^[^ ^35^, ^36^ ^]^

Next, a‐Ni*^δ+^*−PSNS(400) (0< δ ≤1) was obtained through the dehydroxylation activation treatment of Ni−PSNS undergoing in situ reduction in H_2_/N_2_ atmosphere at 400 °C. *Semi‐*in situ XRD profile of a‐Ni*^δ+^*−PSNS(400) still displayed a series of characteristic diffraction peaks of 1:1 type Ni phyllosilicate structure although they became wider and weaker, and no new diffraction peaks had appeared (Figure 1B,C). This implied that the dehydroxylation through moderate temperature reduction could not cause large scale collapse of the main phyllosilicate structure, nor could it lead to the formation of Ni or NiO phase. Furthermore, AC‐HAADF‐STEM showed that a‐Ni*^δ+^*−PSNS(400) inherited the ultrathin nanosheet morphology of Ni−PSNS precursor without the appearance of Ni‐based nanoparticles, and Ni, Si, and O elements remained uniformly distributed within the nanosheet of a‐Ni*^δ+^*−PSNS(400) (Figure 2E,I). Such a result further verified the preservation of the main structure of phyllosilicate in a‐Ni*^δ+^*−PSNS(400) after the dehydroxylation treatment at a moderate reduction temperature. In addition, such an ultrathin 2D nanosheet structure was expected to fully expose Ni*^δ+^* atoms and facilitated efficient mass transfer during the reaction process.^[^ ^37^ ^]^ Notably, compared to a smooth nanosheet surface in Ni−PSNS (Figure 2B), the surface of a‐Ni*^δ+^*−PSNS(400) became obviously rough, and Ni*^δ+^* atoms with an intensified disorder degree of atomic arrangement anchored on the nanosheet can be observed (Figure 2D,F). This may be due to that the dehydroxylation of Ni−O(OH) octahedron layer could not only lead to sub‐outer Ni atoms exposure, but also induce lattice distortion and structural asymmetry and varied atomic distance.^[^ ^38^, ^39^, ^40^ ^]^ Moreover, the extracted line intensity profiles (along the yellow rectangle in Figure 2G,H respectively) showed the increase in image intensity of some originally sub‐outer Ni atoms after the dehydroxylation treatment, further confirming the exposure of Ni*^δ+^* atoms on the nanosheet. Additionally, when the in situ reduction temperature of Ni−PSNS precursor rising to 700 °C, amorphous NiSiO*_x_*‐supported Ni nanoparticles (denoted as Ni/NiSiO*_x_* (700)) were obtained (Figures 1B,C and 2J−L). Correspondingly, *semi‐*in situ XRD profile revealed that the diffraction peaks of phyllosilicate structure disappeared in Ni/NiSiO*_x_* (700), accompanied by the observation of one new weak peak (centered at 44.5°) assigned to an fcc Ni phase (PDF#04‐0850) (Figure 1B,C). Moreover, a very dispersive peak around 21° may be attributed to amorphous SiO*_2_* related species such as NiSiO_x_, which will be further determined and discussed in the subsequent characterizations of EXAFS and XPS. Meanwhile, AC‐HAADF‐STEM and EDS analyses of Ni/NiSiO*_x_* (700) demonstrated that numerous Ni nanoparticles with an average size of ≈5.7 nm were uniformly immobilized onto the amorphous NiSiO*_x_* substrate (Figure 2J−L).

**Figure 3:**
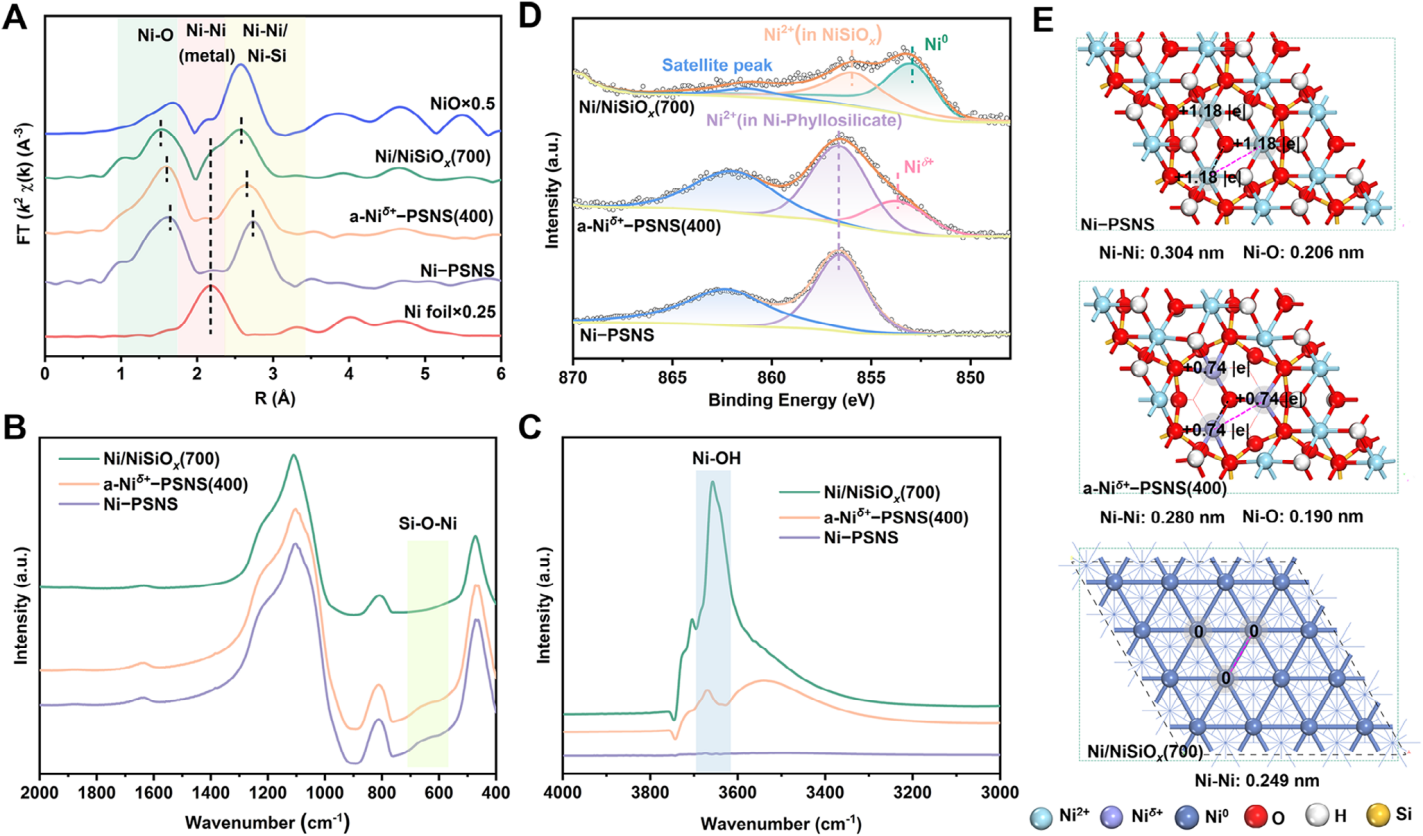
A) Fourier‐transformed *k* ^2^‐weighted EXAFS spectra at Ni *K*‐edge for Ni−PSNS, a‐Ni*^δ+^*−PSNS(400), Ni/NiSiO*_x_* (700), Ni foil and NiO reference. The Fourier‐transforms were not corrected for the phase shift. B) FT‐IR spectra of Ni−PSNS, a‐Ni*^δ+^*−PSNS(400), and Ni/NiSiO*_x_* (700). C) In situ FT‐IR difference spectrum of Ni−PSNS, a‐Ni*^δ+^*−PSNS(400), and Ni/NiSiO*_x_* (700). D) *Semi‐*in situ XPS Ni 2p_3/2_ spectrum of three samples. E) The bond length and Bader charge analysis of three samples by DFT calculation.

Further, the coordinate structure of a‐Ni*^δ+^*−PSNS(400) was investigated by using extended X‐ray absorption fine structure (EXAFS) spectroscopy. From the Fourier‐transform EXAFS spectra at Ni K‐edge (i.e., the R‐space plot, in **Figure** **3**A), after the dehydroxylation treatment at a moderate reduction temperature, the resulting a‐Ni*^δ+^*−PSNS(400) still exhibited the main coordination structure of phyllosilicate (i.e., the first Ni−O shell and the second Ni−Ni/Ni−Si shell of Ni phyllosilicate) similar to that of Ni−PSNS.^[^ ^41^ ^]^ Meanwhile no obvious first Ni−Ni shell of metallic Ni was found in a‐Ni*^δ+^*−PSNS(400). Notably, it can be observed that both the first Ni−O shell distance (1.60 Å) and the second Ni−Ni shell distance (2.65 Å) in a‐Ni*^δ+^*−PSNS(400) were smaller than those in Ni−PSNS (1.64 and 2.74 Å). Such observation was attributed to that the removal of outermost hydroxyl groups led to the Ni−O(OH) octahedral distortions and aggravated structural asymmetry in a‐Ni*^δ+^*−PSNS(400), decreasing the average Ni−O and Ni−Ni distance.^[^ ^42^ ^]^ This was consistent with the trend of Ni−O and Ni−Ni distance changes determined by density functional theory (DFT) calculations (Figure 3E). Additionally, a‐Ni*^δ+^*−PSNS(400) showed a weaker second Ni−Ni shell intensity and a shorter second Ni−Ni shell distance than Ni−PSNS, stemming from the generation of exposed Ni*^δ+^* atoms after the dehydroxylation of the octahedral Ni−O(OH) layer (Figures 3A; S1, Supporting Information).^[^ ^43^, ^44^ ^]^ Thus, these results indicated that after in situ moderate temperature reduction, the outermost hydroxyl groups connected to certain sub‐outer Ni atoms in a‐Ni*^δ+^*−PSNS(400) may be removed, accompanied by the exposure of these sub‐outer Ni atoms. In contrast, the Ni/NiSiO*_x_* (700) sample obtained through in situ high temperature reduction of Ni−PSNS precursor showed obvious unsaturated metallic Ni−Ni coordination (distance: 2.18 Å), suggesting the existence of Ni nanoparticles with small nanoscale in Ni/NiSiO*_x_* (700).^[^ ^43^, ^45^ ^]^ Moreover, the first Ni−O shell and the second Ni−Ni shell were also found in Ni/NiSiO*_x_* (700), and the first Ni−O shell of Ni/NiSiO*_x_* (700) possessed a shorter distance and a weaker intensity than those of the referenced NiO. This was due to the existence of highly dispersed Ni^2+^ species in strong interaction with the SiO_2_ phase in Ni/NiSiO*_x_* (700), which was also consistent with the results of H_2_‐TPR.^[^ ^44^ ^]^

Finally, the characteristic structure and electronic structure of a‐Ni*^δ+^*−PSNS(400) were revealed by using in situ fourier transform infrared (FT‐IR) techniques and *semi‐*in situ X‐ray photoelectron spectroscopy (XPS) respectively. First, FT‐IR spectroscopy from 400 to 2000 cm^−1^ (Figure 3B) and in situ FT‐IR spectroscopy from 3000 to 4000 cm^−1^ (Figure S2, Supporting Information) revealed that Ni−PSNS possessed the absorption bands around 670 and 3650 cm^−1^, assignable to characteristic Si−O−Ni vibration and Ni−OH stretching of 1:1 type Ni phyllosilicate respectively.^[^ ^46^ ^]^ After a moderate temperature reduction, these characteristic bands were still detected in the resulting a‐Ni*^δ+^*−PSNS(400), indicating the preservation of the main structure of phyllosilicate. Meanwhile, through the in situ FT‐IR difference spectrum technique (Figure 3C and see Experimental Section in detail), the intensity difference between the Ni─OH stretching band of a‐Ni*^δ+^*−PSNS(400) and Ni−PSNS obviously increased, attributed to the removal of some outermost hydroxyl groups in the octahedral Ni−O(OH) layer of a‐Ni*^δ+^*−PSNS(400). In contrast, the characteristic vibration bands at 670 cm^−1^ disappeared for Ni/NiSiO*_x_* (700), accompanied by an immense enhancement of the intensity difference of the Ni─OH stretching band, indicating the destruction of Ni phyllosilicate after high temperature reduction (Figure 3B,C).^[^ ^29^, ^47^ ^]^ In addition, the vibration bands at 463, 810, and 1100 cm^−1^ emerge in three samples, belonging to amorphous SiO_2_ (Figure 3B).^[^ ^36^ ^]^ Further, as revealed in *semi‐*in situ deconvoluted Ni 2p_3/2_ XPS spectra (Figure 3D), one dominant deconvoluted peak around 856.6 eV, which was assigned to typical Ni^2+^ species in 1:1 type Ni phyllosilicate, was observed in both Ni−PSNS and a‐Ni*^δ+^*−PSNS(400). Notably, a new small deconvoluted peak at 853.7 eV corresponding to Ni*^δ+^* atoms with low electron density (0< δ ≤1) can be found in a‐Ni*^δ+^*−PSNS(400), stemming from the exposure of some originally sub‐outer Ni atoms after the removal of the outermost hydroxyl group in the octahedral Ni−O(OH) layer.^[^ ^34^, ^41^, ^46^, ^48^, ^49^ ^]^ Meanwhile, no obvious peak attributed to Ni^0^ species was found in a‐Ni*^δ+^*−PSNS(400). In contrast, the resulting Ni/NiSiO*_x_* (700) through in situ high temperature reduction showed two main deconvoluted peaks at 852.9 and 855.9 eV, which were respectively contributed from the metallic Ni^0^ nanoparticles immobilized onto NiSiO*_x_* support and highly dispersed Ni^2+^ species in strong interaction with amorphous SiO_2_.^[^ ^36^, ^44^, ^50^ ^]^

### 2.2 The Moderate‐Low Temperature Catalytic Performance of a‐Ni*^δ+^*−PSNS(400) for RWGS

**Figure 4:**
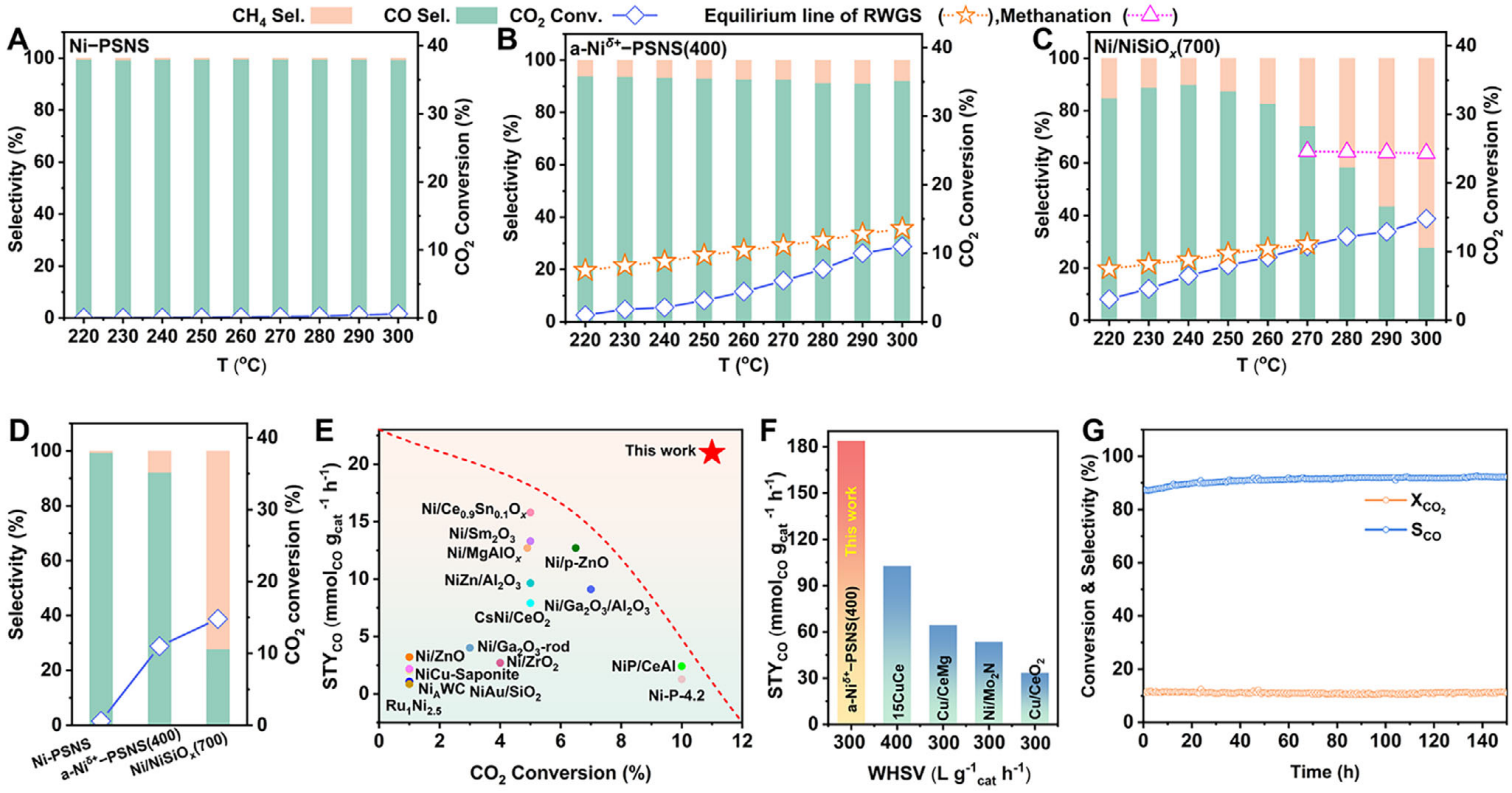
Catalytic performance evolution of A) Ni−PSNS, B) a‐Ni*^δ+^*−PSNS(400), and C) Ni/NiSiO*_x_* (700). MLT‐RWGS reaction conditions: 0.3 g of catalyst, 45:45:10 CO_2_/H_2_/N_2_, and WHSV = 10 000 mL g_catal_^−1^ h^−1^ at 220−300 °C. D) Catalytic performance comparison of three samples at 300 °C. E) CO_2_ conversion vs CO formation rate of a‐Ni*^δ+^*−PSNS(400) and reported prevailing Ni‐based catalysts under MLT‐RWGS reaction conditions (300−350 °C, and atmospheric pressure). F) Comparison of CO formation rates over a‐Ni*^δ+^*−PSNS(400) and recently reported Ni‐based and commonly used Cu‐based catalysts at high WHSV. G) The long‐term stability of a‐Ni*^δ+^*−PSNS tested for 150 h at 300 °C.

Encouraged by the beneficial active structures of as‐prepared a‐Ni*^δ+^*−PSNS(400) (i.e., abundant exposed Ni*^δ+^* atoms with low electron density), we then proceed to investigate whether the a‐Ni*^δ+^*−PSNS(400) had the potential to break through the activity‐selectivity trade‐off and could achieve a boosted catalytic performance for MLT‐RWGS. Catalytic performance evolution of Ni−PSNS, a‐Ni*^δ+^*−PSNS(400), and Ni/NiSiO*_x_* (700) were evaluated under MLT‐RWGS conditions. In the temperature range of 220 to 300 °C, although Ni−PSNS showed high CO selectivity, maintaining above 99%, it had a very poor activity for RWGS, with their CO_2_ conversion not exceeding 1% (**Figure** **4**A). Interestingly, a‐Ni*^δ+^*−PSNS(400) exhibited apparent CO_2_ conversion (≈2%) at the reaction temperature as low as 240 °C (Figure 4B). Meanwhile, their activity elevated significantly with raising the temperature, reaching up to 11.0% of CO_2_ conversion at 300 °C, which was close to the equilibrium conversion at 300 °C. Moreover, although the selectivity of CO in a‐Ni*^δ+^*−PSNS(400) slightly decreased due to the generation of a small amount of methane, they can still remain over 92.0% at all reaction temperatures and MLT‐RWGS always dominated the reaction process. These results implied that abundant exposed Ni*^δ+^* atoms on the nanosheet were critical for enhancing catalytic activity for MLT‐RWGS while effectively inhibiting the occurrence of methanation. In contrast, CO_2_ conversion of Ni/NiSiO*_x_* (700) also showed an increase with the rise of temperature, and ultimately Ni/NiSiO*_x_* (700) achieved a CO_2_ conversion of 14.8%, which was only slightly higher than that over a‐Ni*^δ+^*−PSNS(400) at 300 °C (Figure 4C). However, unlike a‐Ni*^δ+^*−PSNS(400), the reaction selectivity of Ni/NiSiO*_x_* (700) kept on obvious changing with the temperature. The product selectivity significantly changed from 84.7% of CO to 72.3% of CH_4_ as the reaction temperature increased from 220 to 300 °C, and ultimately the reaction was dominated by methanation at 300 °C. This was likely due to that the formation of Ni^0^ nanoparticles in Ni/NiSiO*_x_* (700) greatly favored CO hydrocracking and the resulting methanation.^[^ ^8^, ^51^ ^]^

Next, catalytic performance data at 300 °C of three catalysts (Figures 4D; S3, Supporting Information) indicated that a‐Ni*^δ+^*−PSNS(400) not only gave excellent catalytic activity and selectivity toward MLT‐RWGS simultaneously, but also achieved a maximum CO formation rate (21.0 mmol_CO_ h^−1^ g_cat_^−1^). Furthermore, the TOF values for CO formation and CH_4_ formation were evaluated. It was found that a‐Ni*^δ+^*−PSNS(400) exhibited a larger TOF value of product CO formation (0.023 S^−1^ vs 0.019 S^−1^) while a significantly lower TOF value of by‐product CH_4_ formation (0.002 S^−1^ vs 0.048 S^−1^) than those of Ni/NiSiOx(700). This result implied that exposed Ni*^δ+^* atoms on a‐Ni*^δ+^*−PSNS(400) unlike Ni^0^ nanoparticles can effectively catalyze CO_2_ reduction into CO while greatly inhibited the deep hydrogenation of CO into CH_4_, likely owing to a nearly single atom‐dispersed geometric configuration and low electron density of Ni*^δ+^* atoms. Additionally, the apparent activation energies (*E*a) for MLT‐RWGS reaction were measured for a‐Ni*^δ+^*−PSNS(400) and Ni/NiSiO*_x_* (700) in the temperature range of 220 to 300 °C (Figure S4, Supporting Information). The a‐Ni*^δ+^*−PSNS(400) had a lower *E*a compared with Ni/NiSiO*_x_* (700) (59.0 vs 72.0 kJ mol^−1^). Moreover, as shown in Figure 4E and Table S1 (Supporting Information), owing to the presence of an activity‐selectivity trade‐off, it was very challenging for previously reported Ni‐based catalysts to achieve the combination of high activity and high selectivity as well as outstanding CO formation rate in MLT‐RWGS.^[^ ^9^, ^12^, ^14^, ^26^ ^]^ However, a‐Ni^δ+^−PSNS(400) successfully broke through such activity‐selectivity trade‐off, with an outstanding CO selectivity of 92.0% while maintaining a high CO_2_ conversion of 11.0% at 300 °C, and achieving an impressive CO formation rate of 21.0 mmol_CO_ h^−1^ g_cat_^−1^, which outdistanced the CO formation rate of all prevailing Ni‐based catalysts of MLT‐RWGS previously reported, and superior to recently reported many Cu‐based and noble‐metal‐based catalysts for MLT‐RWGS (Figure 4D,E; Table S1, Supporting Information). Besides, a‐Ni*^δ+^*−PSNS(400) catalyzed MLT‐RWGS was performed at 300 °C with various weight hourly space velocity (WHSV). When the WHSV increased from 10 000 to 300 000 mL g_catal_^−1^ h^−1^, the selectivity of CO elevated from 92.0% to 95.7% and the CO formation rate also significantly increased from 21.0 to 184.0 mmol_CO_ h^−1^ g_cat_^−1^ (Figure S5, Supporting Information). Especially, even at high WHSV, a‐Ni*^δ+^*−PSNS(400) exhibited catalytic performance for MLT‐RWGS far superior to recently reported Ni‐based and commonly used Cu‐based catalysts, with an outstanding CO formation rate of 184.0 mmol_CO_ h^−1^ g_cat_^−1^ at WHSV of 300 000 mL g_catal_^−1^ h^−1^ (Figure 4F; Table S1, Supporting Information).

Next, the catalytic performances of a series of Ni‐based catalysts obtained from ultrathin Ni‐phyllosilicate nanosheet treated with different in situ reduction temperatures were also evaluated (the structural characterizations and catalytic performances of these Ni‐based catalysts, as well as related detailed discussions, were presented in Figures S6−S11 and Table S2, and Supporting Information). It was found that as the in situ reduction temperature increased from 300 to 700 °C, the CO formation rate of the obtained catalyst changed in a volcanic manner, with a maximum CO formation rate present in a‐Ni*^δ+^*−PSNS(400) (Figure S11, Supporting Information). Besides, a‐Ni^δ+^‐PSNS(400‐15) catalyst with higher Ni loading (about Ni loading of 15%) was also prepared and its catalytic performance for MLT‐RWGS at 300 °C was evaluated (its structural characterizations and catalytic performances, as well as related detailed discussions, were presented in Figure S12 and Supporting Information, Supporting Information). The CO_2_ conversion of a‐Ni^δ+^‐PSNS(400‐15) increased while its CO selectivity decreased compared to a‐Ni^δ+^‐PSNS(400) (12.3% vs 11.0% for CO_2_ conversion and 79.4% vs 92.0% for CO selectivity), and correspondingly methanation was further driven, resulting in a slight decrease in CO formation rate (19.6 mmol_CO_ h^−1^ g_cat_^−1^) (Figure S12D, Supporting Information). Finally, the long‐term stability of a‐Ni*^δ+^*−PSNS(400) was investigated under MLT‐RWGS conditions (Figures 4G; S13, Supporting Information). The CO_2_ conversion can be maintained at 11.0% with above 92.0% of CO selectivity after 150 h, and the used a‐Ni^δ+^−PSNS(400) basically maintained the structure of Ni^δ+^ atoms anchored on ultrathin Ni‐phyllosilicate nanosheet without obvious production of Ni^0^ species. This indicated that a‐Ni*^δ+^*−PSNS(400) possessed both excellent catalytic performance and high stability for MLT‐RWGS.

### 2.3 Catalytic Mechanism of a‐Ni*^δ+^*−PSNS(400) for MLT‐RWGS

**Figure 5:**
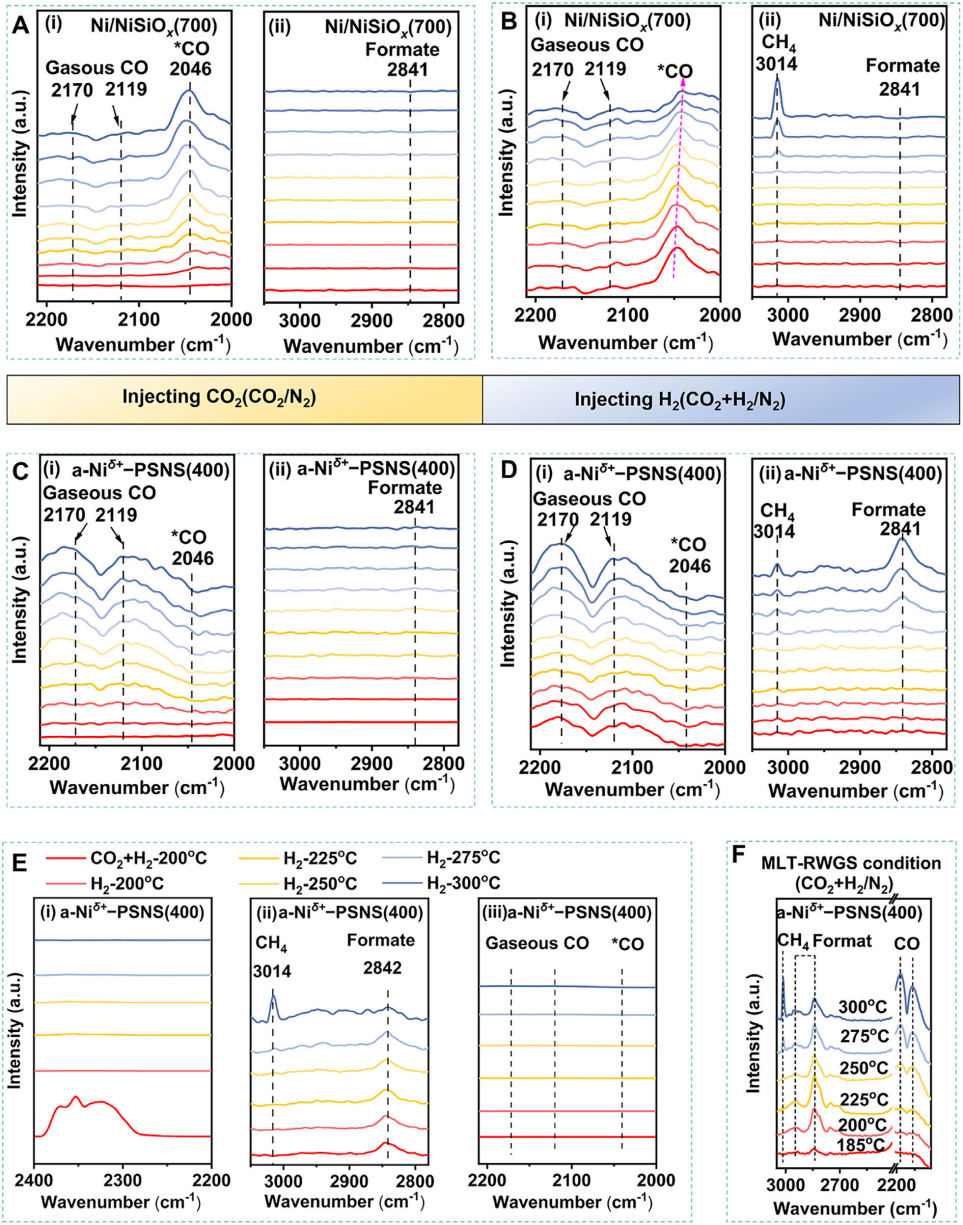
In situ DRIFTS spectra for Ni/NiSiO*_x_* (700) under A) CO_2_/N_2_ and B) subsequent injecting H_2_/N_2_ at 300 °C. In situ DRIFTS spectra for a‐Ni*^δ+^*−PSNS(400) under C) CO_2_/N_2_ and D) subsequent injecting H_2_/N_2_ at 300 °C. E) In situ DRIFTS spectra with programmed heating for identifying the conversion path of formate intermediates on the a‐Ni*^δ+^*−PSNS(400). G) In situ DRIFTS spectra with programmed heating for a‐Ni*^δ+^*−PSNS(400) under MLT‐RWGS conditions (CO_2_ + H_2_/N_2_).

In situ diffuse reflectance infrared Fourier transform spectroscopy (DRIFTS) was first performed to identify the potential catalytic mechanism of a‐Ni*^δ+^*−PSNS(400) for MLT‐RWGS. Specifically, the system was first injected with CO_2_/N_2_ gas for 30 min and then injected with H_2_/N_2_ gas for another 30 min, and the system was measured by DRIFTS with a time resolution mode (see Experimental Section for details). For Ni/NiSiO*_x_* (700) as a comparative study (**Figures** **5**A–I; S14A, Supporting Information), the first injection into CO_2_ showed the rapid appearance of an adsorbed CO signal around 2046 cm^−1^ along with the gaseous CO peaks at 2170 and 2119 cm^−1^.^[^ ^52^ ^]^ Moreover, no peaks at 2841 cm^−1^ associated with formate species (^*^HCOO) were observed, while carboxyl species (^*^COOH) were also absent (Figures 5A‐ii; S14A, Supporting Information).^[^ ^13^, ^53^, ^54^, ^55^, ^56^, ^57^, ^58^ ^]^ These results suggested that CO_2_ can be activated‌ on Ni^0^ species of Ni/NiSiO*_x_* (700) in the absence of H_2_, with reducing CO_2_ to CO directly and forming oxidized Ni^2+^ species concomitantly, which was most likely involved a redox mechanism rather than the associative intermediate pathway.^[^ ^7^, ^59^ ^]^ After injecting into H_2_/N_2_, the gaseous CO can still be observed, and there was still no appearance of formate and carboxyl species (Figures 5B; S14B, Supporting Information). Notably, with the time on stream, adsorbed CO signal gradually decreased and underwent redshift, accompanied by the significant appearance of the CH_4_ signal at 3014 cm^−1^ (Figures 5B‐ii; S14B, Supporting Information).^[^ ^60^ ^]^ This was attributed to the reduction of Ni^2+^ species oxidized by CO_2_ back to Ni^0^ under H_2_/N_2_ and subsequent easy hydrogenation of adsorbed CO on Ni^0^ to CH_4_.^[^ ^61^ ^]^ Additionally, in situ DRIFTS spectra of Ni/NiSiO*_x_* (700) during reaction conditions (CO_2_ + H_2_) revealed the generation of a weak CO signal accompanied by a strong CH_4_ signal without a signal of formate, further confirming easy hydrogenation of adsorbed CO on Ni^0^ sites of Ni/NiSiO*_x_* (700) to CH_4_ (Figure S15, Supporting Information). This was in line with the MLT‐RWGS results, where Ni/NiSiO*_x_* (700) showed a low product CO yield while significant by‐product CH_4_. Therefore, Ni/NiSiO*_x_* (700) could catalyze MLT‐RWGS through a redox mechanism, but a large amount of product CO adsorbed on Ni^0^ sites would be consumed and further hydrogenated into by‐product CH_4_.

By contrast, exposure of a‐Ni*^δ+^*−PSNS(400) to a CO_2_/N_2_ flow also showed the rapid appearance of only the gaseous CO peaks and no generation of formate and carboxyl species as revealed by in situ DRIFTS spectra, which was likely due to the presence of Ni*^δ+^* species, which can reduce CO_2_ to CO directly and form Ni^2+^ species concomitantly (Figures 5C; S16A, Supporting Information).^[^ ^7^ ^]^ These, thus, indicated that the activation of CO_2_ into CO on the a‐Ni*^δ+^*−PSNS(400) may also occur through a redox mechanism. After H_2_/N_2_ injection, the gaseous CO signal gradually increased with the time on stream, while a new feature corresponding to formate species which was absent with H_2_ injecting on the surface of Ni/NiSiO*_x_* (700) was also observed at 2841 cm^−1^ and gradually elevated, and accompanied by the appearance of a weak CH_4_ signal (Figures 5D; S16B, Supporting Information).^[^ ^62^ ^]^ Meanwhile, no peaks attributed to carboxyl species were found. These implied that in addition to the redox mechanism, CO_2_ may be also activated through an associative H_2_ pathway with formate as an intermediates (i.e., hydrogen‐mediated CO_2_ activation via formate intermediates), and the resulting formate intermediates may be further converted into CO or/and CH_4_.^[^ ^62^, ^63^ ^]^ Further, under H_2_/N_2_ the conversion path of formate intermediates on the a‐Ni*^δ+^*−PSNS(400) was identified by in situ DRIFTS spectra with programmed heating (Figure 5E). Specifically, the CO_2_ + H_2_/N_2_ mixed gas was first injected into the reaction cell of in situ DRIFTS spectroscopy at 200 °C; after CO_2_ was completely consumed and converted to formate species (Figure 5E‐i), the system programed temperature rise under H_2_/N_2_ to observe the conversion of formate species (Figure 5E‐ii,iii). There was almost no change in the formate signal and no significant CO or methane signal was observed when programed heating from 200 to 275 °C (Figure 5E‐ii,iii). Notably, after the temperature rose to 300 °C, formate species reduced, accompanied by CH_4_ production but no CO production. This demonstrated that the activation of CO_2_ into CO on the a‐Ni^δ+^−PSNS(400) was achieved through a redox mechanism rather than an associative mechanism with formate as an intermediate, while the slight amount of by‐product CH_4_ in a‐Ni*^δ+^*−PSNS(400) catalyzed MLT‐RWGS was likely converted from formate intermediates generated by hydrogen‐assisted partial CO_2_ activation rather than from product CO hydrogenation like Ni/NiSiO*_x_* (700). Correspondingly, the formate conversion simulated in a tube furnace coupled with a MASS spectrometer further confirmed that the formate formed on a‐Ni^δ+^−PSNS(400) surface was ultimately converted to CH_4_ rather than CO (Figure S17, Supporting Information), meanwhile, the temperature programmed surface reaction (TPSR) also revealed that it was difficult to trigger the hydrogenation of CO to CH_4_ on a‐Ni*^δ+^*−PSNS(400) under MLT‐RWGS conditions (Figure S18, Supporting Information). Additionally, as revealed by in situ DRIFTS spectra with programmed heating under MLT‐RWGS conditions (CO_2_ + H_2_/N_2_), the conversion of CO_2_ into CO through a redox mechanism on the a‐Ni*^δ+^*−PSNS(400) surface was more favorable than hydrogen‐mediated activation of CO_2_ into CH_4_, thereby enabling MLT‐RWGS to dominate, meanwhile the conversion of formate intermediates into CH_4_ on a‐Ni*^δ+^*−PSNS(400) rather than CO was also confirmed again (Figure 5F).

**Figure 6:**
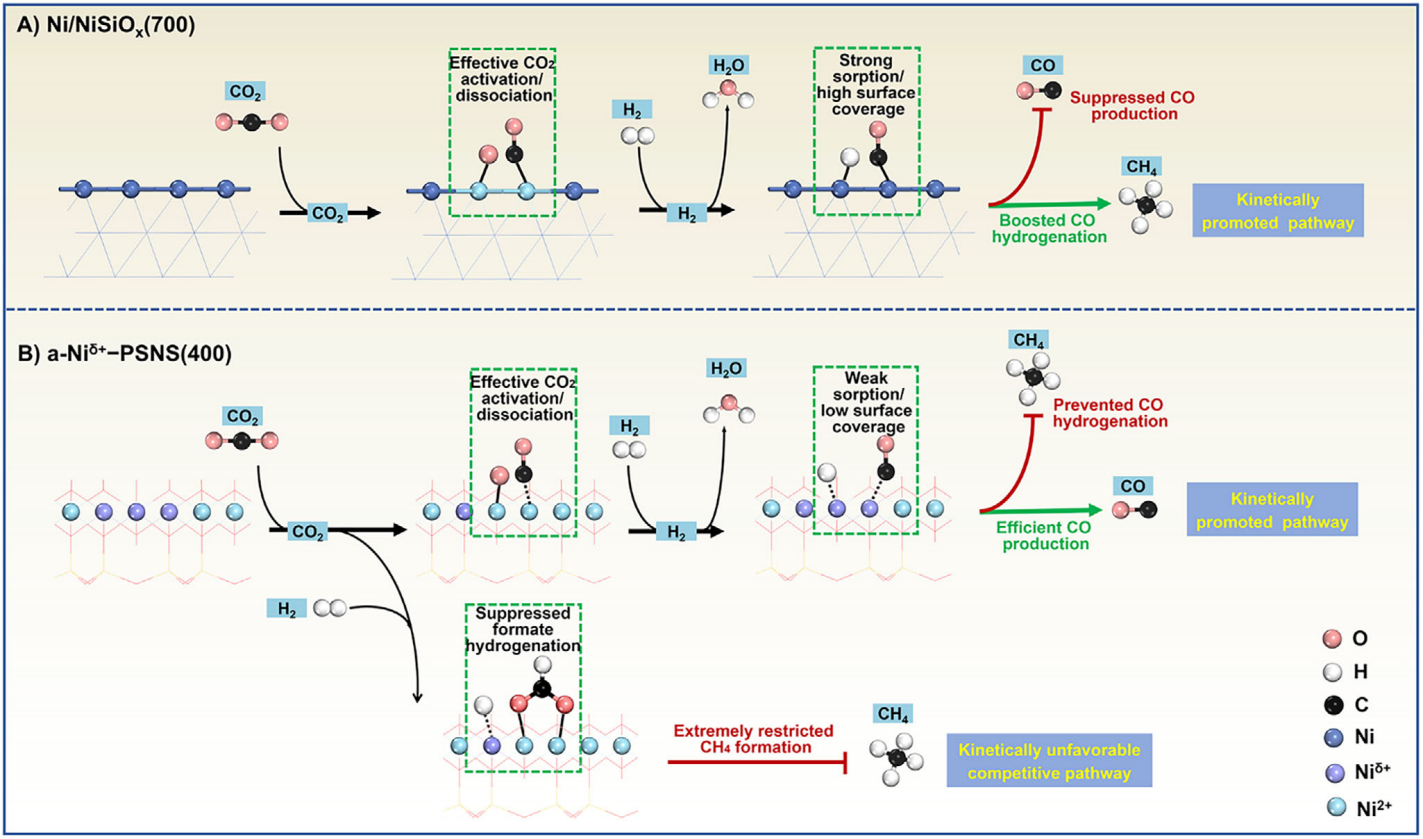
Schematic illustration of the catalytic mechanism of A) Ni/NiSiO*_x_* (700) and B) a‐Ni*^δ+^*−PSNS(400) for MLT‐RWGS.

According to the previous reports, the product CO selectivity in MLT‐RWGS of Ni‐based catalysts, especially during high CO_2_ conversion, was seriously discount due to their high capacity of CO activation and C−O bond cleavage and the resulting significant CO methanation.^[^ ^64^, ^65^ ^]^ Similarly, the Ni/NiSiO*_x_* (700) in this work had not been spared either since its Ni^0^ sites boosted CO chemisorption and hydrogen dissociation and surface coverage and exacerbated CO methanation (Figures 4C and 5A,B and **6**A; S15, S18−S22). In contrast, a‐Ni*^δ+^*−PSNS(400) almost avoided product CO hydrocracking into by‐product CH_4_ at a high CO_2_ conversion, breaking an irreconcilable activity‐selectivity trade‐off and achieving outstanding CO yield (Figures 4B and 6B). This was likely attributed to abundant exposed Ni*^δ+^* atoms with low electron density on a‐Ni*^δ+^*−PSNS(400). On the one hand, the ultrathin nanosheet structure enabled abundant Ni*^δ+^* atoms to fully expose and disperse, boosting atom‐utilization efficiency and atom‐synergistic effects, endowing them with high catalytic activity. Ni*^δ+^* atoms can efficiently convert CO_2_ into CO through a redox mechanism (Figure 5C), while adjacent Ni*^δ+^* atoms can effectively dissociate H_2_ to promote the redox cycle (Figure S21, Supporting Information). On the other hand, Ni*^δ+^* atoms had a relatively low electron density compared to Ni^0^ sites, displaying unique electronic effects (Figure 3D,E). The low electron density of Ni*^δ+^* atoms extremely weakened their chemical adsorption of CO, preventing the ^*^CO intermediate hydrocracking and CO methanation, and also accelerating Ni*^δ+^* sites to start a new turnover and MLT‐RWGS catalysis (Figures 5C,D; S18−S20, Supporting Information). Moreover, chemisorption of dissociated H by Ni*^δ+^* atoms was weak and led to low hydrogen surface coverage, which was also beneficial for suppressing CO hydrocracking (Figure S22, Supporting Information). Besides, Ni*^δ+^* sites could preferentially activate CO_2_ hydrogenation to CO while effectively suppress competitive CO_2_ hydrogenation to CH_4_, further ensuring MLT‐RWGS to dominate (Figure 5F).

**Figure 7:**
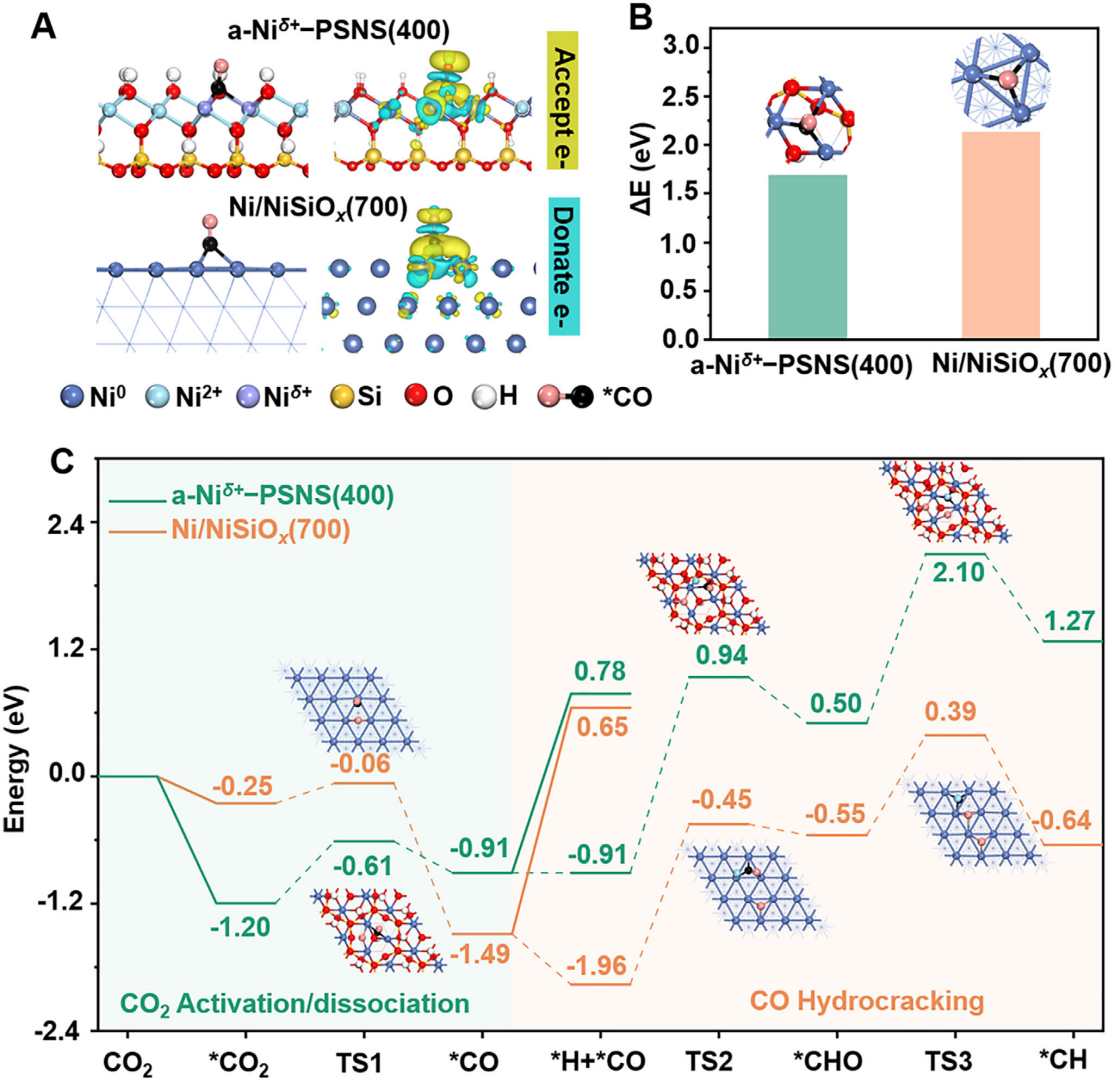
A) The charge density difference for CO adsorption on a‐Ni*^δ+^*−PSNS(400) and Ni/NiSiO*_x_* (700). B) Calculated desorption energies of ^*^CO on a‐Ni*^δ+^*−PSNS(400) and Ni/NiSiO*_x_* (700). C) Potential energy diagrams of the direct dissociation of CO_2_ into CO through redox mechanism and the possibility of subsequent CO hydrocracking on the a‐Ni*^δ+^*−PSNS(400) and Ni/NiSiO*_x_* (700) surfaces.

Besides, density functional theory (DFT) calculations were performed to further reveal the catalytic mechanism of a‐Ni*^δ+^*−PSNS(400) for MLT‐RWGS. Weakening chemisorption of the ^*^CO intermediate on catalyst surfaces was the key to inhibiting ^*^CO hydrocracking, while the electronic effect of catalysts largely determined their chemisorption strength of ^*^CO.^[^ ^16^, ^66^, ^67^ ^]^ The charge density difference for CO adsorption on a‐Ni*^δ+^*−PSNS(400) and Ni/NiSiO*_x_* (700) was first calculated to reveal their electronic effects, where the charge density distributions of both a‐Ni*^δ+^*−PSNS(400) and Ni/NiSiO*_x_* (700) surfaces with adsorbed ^*^CO were analyzed by comparing the electron charge donation/acceptance status (**Figure** **7**A). It was found that electron charge donation was observed on both Ni*^δ+^* sites of a‐Ni*^δ+^*−PSNS(400) and Ni^0^ sites of Ni/NiSiO*_x_* (700), accompanied by electron charge accumulation between *CO and the sites. However, the electron charge transformation from Ni*^δ+^* sites to adsorbed CO was significantly less than that from Ni^0^ sites, which would lead to that a‐Ni*^δ+^*−PSNS(400) exhibited significantly weaker adsorption of ^*^CO intermediate compared with Ni/NiSiO*_x_* (700).^[^ ^68^ ^]^ Correspondingly, it was observed that the desorption energy of ^*^CO (1.69 eV) on the a‐Ni*^δ+^*−PSNS(400) was lower than that on the Ni/NiSiO*_x_* (700) (2.13 eV), confirming a much weaker adsorption of ^*^CO on a‐Ni*^δ+^*−PSNS(400) (Figure 7B). This was also consistent with the experimental results of in situ DRIFTS spectra (Figures 5; S19, Supporting Information) and CO−TPD (Figure S20, Supporting Information). Such electronic effect and weak adsorption of a‐Ni*^δ+^*−PSNS(400) to ^*^CO were derived from unique electronic structure of Ni*^δ+^* sites: XPS results (Figure 3D) and Bader charge analysis (Figure 3E) confirmed that Ni*^δ+^* sites possessed lower electron density compared with Ni^0^ sites; and the reduced electron density of Ni*^δ+^* sites could decrease electron transformation from Ni*^δ+^* sites to adsorbed CO (as shown in Figure 7A) and down‐regulate d−*π* electron feedback from Ni*^δ+^* sites to antibonding orbital (π^*^) of adsorbed CO;^[^ ^69^, ^70^ ^]^ thus resulted in weakening of Ni*^δ+^* ─C bond while strengthening of C─O bond, ultimately boosting CO desorption and inhibiting CO hydrocracking. In addition, such enhanced CO desorption could also accelerate Ni*^δ+^* sites to start a new turnover and promote the RWGS catalysis.

Potential energy diagrams of the direct dissociation of CO_2_ into CO through redox mechanism and the possibility of subsequent CO hydrocracking was further investigated on the a‐Ni*^δ+^*−PSNS(400) and Ni/NiSiO*_x_* (700) surfaces (Figure 7C, and the corresponding configurations were summarized in Figures S23 and S24, Supporting Information). As we can see, both a‐Ni*^δ+^*−PSNS(400) (0.59 eV) and Ni/NiSiO*_x_* (700) (0.19 eV) showed relatively low energy barrier for the dissociation of CO_2_ into ^*^CO and ^*^O, suggesting that the activation of CO_2_ on their surfaces were kinetically feasible. However, the desorption energy of CO (1.69 eV) on the a‐Ni*^δ+^*−PSNS(400) surface was lower than the energy barrier of CO hydrogenation into ^*^CHO (1.85 eV), indicating that further CO hydrogenation (i.e., CO methanation) was inhibited, while favoring MLT‐RWGS process and gaseous CO production. Moreover, the subsequent CO hydrocracking on a‐Ni*^δ+^*−PSNS(400) surface was both thermodynamically and kinetically unfavorable to carry out. In contrast, the high CO desorption energy (2.13 eV) on the Ni/NiSiO*_x_* (700) surface facilitated further hydrogenation of CO to ^*^CHO (1.51 eV), and the following CO hydrocracking on Ni/NiSiO*_x_* (700) surface was relatively more advantageous to carry out, thereby promoting CO methanation and producing the by‐product CH_4_. Therefore, MLT‐RWGS was preferred to efficiently produce CO on a‐Ni*^δ+^*−PSNS(400) in contrast with the adverse CO methanation prevailing on Ni/NiSiO*_x_* (700). In addition, CO_2_ hydrogenation into CO through the H‐assisted ^*^COOH mechanism was also discussed by DFT calculation. Correspondingly, the resulting potential energy diagrams and configurations were summarized in Figures S25−S27 (Supporting Information). As can be seen from potential energy diagrams of H‐assisted ^*^COOH mechanism, the energy barrier of the ^*^CO_2_ hydrogenation to ^*^COOH was sufficient higher than that of the subsequent ^*^COOH dissociation to ^*^CO and ^*^OH on a‐Ni^δ+^−PSNS(400) (1.54 eV vs 0.18 eV) and Ni/NiSiOx(700) (0.79 eV vs 0.31 eV). This indicated that the rate‐determining step of the H‐assisted ^*^COOH pathway were the formation of ^*^COOH. Meanwhile, energy barriers of ^*^CO_2_ hydrogenation to ^*^COOH were significantly higher than that of ^*^CO_2_ direct dissociation into ^*^CO and ^*^O (1.54 vs 0.59 eV on a‐Ni^δ+^−PSNS(400) and 0.79 vs 0.19 eV on Ni/NiSiOx(700)). Therefore, the redox mechanism was energetically more favorable than the H‐assisted ^*^COOH mechanism for the RWGS process in this system.

## 3 Conclusion

In summary, we developed a novel supported Ni‐based catalyst, consisting of abundant Ni*^δ+^* atoms with low electron density anchored in situ on ultrathin Ni‐phyllosilicate nanosheet, via a convenient two‐step procedure including construction of 1:1 type nickel phyllosilicate nanosheet precursor and subsequent in situ reduction at moderate temperature. Unlike other Ni‐based catalysts, a‐Ni*^δ+^*−PSNS(400) brake through an irreconcilable activity‐selectivity trade‐off and achieved a simultaneous high CO_2_ conversion and high CO selectivity. The resulting catalytic performance outdistanced all prevailing Ni‐based catalysts for MLT‐RWGS. Experimental and theoretical studies demonstrated that a‐Ni*^δ+^*−PSNS(400) provided abundant Ni*^δ+^* atoms with unique geometric/electronic effects and thereby exhibited highly favorable catalytic properties for MLT‐RWGS. Their ultrathin nanosheet structure enabled abundant anchored Ni*^δ+^* atoms to fully expose and disperse, enhancing atom‐utilization efficiency and atom‐synergistic effects. Accordingly, Ni*^δ+^* atoms can efficiently reduce CO_2_ into CO through a redox mechanism; meanwhile, adjacent Ni*^δ+^* atoms can activate H_2_, promoting the redox cycle. Moreover, the low electron density of Ni^δ+^ atoms extremely weakened their chemical adsorption of CO, preventing further ^*^CO intermediate hydrocracking and subsequent CO methanation, and also accelerating Ni^δ+^ sites to start a new turnover and MLT‐RWGS catalysis. Meanwhile, Ni^δ+^ atoms also effectively suppressed competitive CO_2_ hydrogenation to CH_4_. This work provided new insights into the design of active microstructures of high‐performance Ni‐based catalysts for synchronous high activity‐selectivity.

## Conflict of Interest

The authors declare no conflict of interest.

## Supporting information



Supporting Information

## Data Availability

The data that support the findings of this study are available from the corresponding author upon reasonable request.

## References

[1] D. Gilfillan , G. Marland , Earth Syst. Sci. Data 2021, 13, 1667.

[2] J. L. Penn , C. Deutsch , Science 2022, 376, 524. 35482875 10.1126/science.abe9039

[3] W. Wang , S. Wang , X. Ma , J. Gong , Chem. Soc. Rev. 2011, 40, 3703. 21505692 10.1039/c1cs15008a

[4] W. Zhou , K. Cheng , Y. Wang , Chem. Soc. Rev. 2019, 48, 3193. 31106785 10.1039/c8cs00502h

[5] H. M. T. Galvis , K. P. d. Jong , Science 2012, 335, 835.22344440

[6] H. X. Liu , S. Q. Li , C. J. Jia , Nat. Commun. 2022, 13, 867. 35165303 10.1038/s41467-022-28476-5 PMC8844362

[7] C. Hansen , W. Zhou , E. Brack , Y. Wang , C. Wang , J. Paterson , J. Southouse , C. Copéret , J. Am. Chem. Soc. 2024, 146, 27555. 39347826 10.1021/jacs.4c08517

[8] C. Vogt , E. Groeneveld , B. M. Weckhuysen , Nat. Catal. 2018, 1, 127.

[9] S. Zou , Y. Liang , X. Zhang , Q. Gu , L. Wang , H. Sun , X. Liao , J. Huang , A. R. Masri , Angew. Chem. Int. Ed. 2024, 64, 202412835. 10.1002/anie.202412835 39172117

[10] T. S. Galhardo , A. H. Braga , B. H. Arpini , J. Szanyi , R. V. Gonçalves , B. F. Zornio , C. R. Miranda , L. M. Rossi , J. Am. Chem. Soc. 2021, 143, 4268. 33661617 10.1021/jacs.0c12689

[11] S. Zhang , H. Ma , L. Jia , Z. Zhang , X. Li , S. Dang , Y. Huang , Y. Tian , W. Tu , Y.i‐F. Han , Appl. Catal. B Environ. 2025, 361, 124646.

[12] D. Ye , Z. Wu , T. Wang , R. Zhu , Y. Feng , J. Lei , Y. Tian , Z. Zou , H. Wu , C. Cheng , S. Tang , S. Li , Adv. Mater. 2025, 2504431. 10.1002/adma.202504431 40304145

[13] C. X. Wang , H. X. Liu , C. J. Jia , Nat. Commun. 2024, 15, 8290. 39333511 10.1038/s41467-024-52547-4 PMC11437244

[14] L. Lin , J. Liu , X. Liu , Z. Gao , N. Rui , S. Yao , F. Zhang , M. Wang , C. Liu , L. Han , F. Yang , S. Zhang , X.‐D. Wen , S. D. Senanayake , Y. Wu , X. Li , J. A. Rodriguez , D. Ma , Nat. Commun. 2021, 12, 6978. 34848709 10.1038/s41467-021-27116-8 PMC8632928

[15] L. Lin , X. Tang , Y. Wang , J. Zhang , C. Yu , M. Cheng , S. Yang , X. Yang , L. Liu , L. Han , Y. Xu , C. Song , Angew. Chem. Int. Ed. 2025, 64, 202511453. 10.1002/anie.202511453 40626988

[16] S. Lin , Q. Wang , M. Li , Z. Hao , Y. Pan , X. Han , X. Chang , S. Huang , Z. Li , X. Ma , ACS Catal. 2022, 12, 3346.

[17] J. Zhao , X. Liu , D. Su , ACS Catal. 2024, 14, 3158.

[18] R. Ye , L. Ma , X. Hong , T. R. Reina , W. Luo , L. Kang , G. Feng , R. Zhang , M. Fan , R. Zhang , J. Liu , Angew. Chem. Int. Ed. 2023, 63, 202317669. 10.1002/anie.202317669 38032335

[19] B. Zhao , B. Yan , J. G. Chen , Chem. Commun. 2018, 7354. 10.1039/c8cc03829e 29911232

[20] R. A. V. Santen , Acc. Chem. Res. 2009, 42, 57. 18986176 10.1021/ar800022m

[21] J. Li , Y. Lin , X. Bao , ACS Catal. 2019, 9, 6342.

[22] D. Wang , Z. Yuan , X. Wu , W. Xiong , J. Ding , Z. Zhang , W. Huang , ACS Catal. 2023, 13, 7132.

[23] X. Yuan , T. Pu , M. Gu , M. Zhu , J. Xu , ACS Catal. 2021, 11, 11966.

[24] Y. Wang , J. Chen , L. Chen , Y. Li , ACS Catal. 2023, 13, 3735.

[25] L. R. Winter , B. Yan , J. G. Chen , Appl. Catal. B Environ. 2018, 224, 442.

[26] X. Wei , G. Johnson , Y. Ye , M. Cui , S.‐W. Yu , Y. Ran , J. Cai , Z. Liu , X. Chen , W. Gao , P. J. L. Bean , W. Zhang , T. Y. Zhao , F. A. Perras , E. J. Crumlin , X.u Zhang , R. J. Davis , Z. Wu , S. Zhang , J. Am. Chem. Soc. 2023, 145, 14298. 37345939 10.1021/jacs.3c02739

[27] M. M. Millet , G. Algara Siller , E. Frei , J. Am. Chem. Soc. 2019, 141, 2451. 30640467 10.1021/jacs.8b11729 PMC6728101

[28] Z. Bian , S. Kawi , Catal. Today 2020, 339, 3.

[29] X. Kong , Y. Zhu , H. Zheng , X. Li , Y. Zhu , Y.‐W. Li , ACS Catal. 2015, 5, 5914.

[30] H. Dong , Q. Liu , ACS Sus. Chem. Eng. 2020, 8, 6753.

[31] E. Hondo , T. Z. H. Gani , M. Kosari , S. Xi , Bella , J. Ashok , J. Y. Tan , T. Wang , H. Bian , K. H. Lim , L. Chen , J. Chang , A. Borgna , S. Kawi , ACS Sus. Chem. Eng. 2023, 11, 8786.

[32] M. Li , Z. Li , Q. Lin , J. Cao , F. Liu , M. H. Wai , S. Kawi , Catal. Today 2022, 402, 319.

[33] Z. Li , Y. Kathiraser , J. Ashok , U. Oemar , S. Kawi , Langmuir 2014, 30, 14694. 25397692 10.1021/la503340s

[34] E.‐J. Kim , Y. Woo Kim , Y. Cho , S. Kweon , M. Bum Park , C.‐H. Shin , H.‐K. Min , K. An , Chem. Eng. J. 2024, 485, 149871.

[35] D. Wang , J. Liu , H. Li , Q. Liu , Y. Cheng , X. Fan , P. Liang , Appl. Catal. B Environ. 2023, 327, 122452.

[36] R.‐P. Ye , W. Gong , Z. Sun , Q. Sheng , X. Shi , T. Wang , Y. Yao , J. J. Razink , L. Lin , Z. Zhou , H. Adidharma , J. Tang , M. Fan , Y.‐G. Yao , Energy 2019, 188, 116059.

[37] T. Cui , Y.‐P. Wang , T. Ye , J. Wu , Z. Chen , J. Li , Y. Lei , D. Wang , Y. Li , Angew.Chem. Int. Ed. 2022, 61, 202115219. 10.1002/anie.202115219 34994045

[38] D. Wu , T. Y. Longfei , Nat. Commu. 2015, 16, 726.

[39] M. Aral , S.‐L. Guo , Y. Nishiyama , J. Catal. 1992, 135, 638.

[40] B. Chen , X. Ouyang , Y. Qian , Green Chem. 2022, 24, 846.

[41] Z. Luo , X. Ge , D. Fang , X. Xu , D. Zhang , Y. Cao , X. Duan , W. Li , J. Zhou , X. Zhou , J. Catal. 2024, 434, 115528.

[42] Y. Zhao , X. Jia , T. Zhang , J. Am. Chem. Soc. 2016, 138, 6517. 27159825 10.1021/jacs.6b01606

[43] H. Chen , S. He , X. Cao , S. Zhang , M. Xu , M. Pu , D. Su , M. Wei , D. G. Evans , X. Duan , Chem. Mater. 2016, 28, 4751.

[44] K. Feng , S. Qian , Z. Zhang , Z. Li , X. Sun , Y.i Cheng , B. Yan , Chem. Eng. J. 2023, 465, 142808.

[45] H. Chen , S. He , X. Duan , ACS Catal. 2017, 7, 2735.

[46] E. Soghratia , T. K. C. Onga , A. Borgnaa , Appl. Catal. B Environ. 2018, 235, 130.

[47] Q. Zhang , Z. Jiang , Y. Zhang , X. Xu , Y. Yang , Y. Qin , L. Song , Y. Mei , Y. Zu , Appl. Catal. B Environ. 2025, 362, 124722.

[48] C. Wang , X. Hai , J. Bai , Y. Shi , L. Jing , H. Shi , Z. Chen , Y. Zhao , Chem. Eng. J. 2024, 488, 150723.

[49] N. Liu , B. Chen , N. Zheng , ACS Catal. 2023, 13, 7347.

[50] B. Zhao , Z. Chen , Y. Chen , X. Ma , Int. J. Hydrogen Energy 2017, 42, 27073.

[51] H. Zheng , W. Liao , J. Ding , F. Xu , A. Jia , W. Huang , Z. Zhang , ACS Catal. 2022, 12, 15451.

[52] F. C. Meunier , Catal. Today 2023, 423, 113863.

[53] J. K. Li , J. P. Dong , S. S. Liu , Y. Hua , X.‐L. Zhao , Z. Li , S.‐N. Zhao , S.‐Q. Zang , R. Wang , Angew.Chem. Int. Ed. 2024, 63, 202412144. 10.1002/anie.202412144 39169221

[54] X. Wan , Y. Li , Y. Chen , J. Ma , Y.‐A. Liu , E.‐D. Zhao , Y. Gu , Y. Zhao , Y. Cui , R. Li , D. Liu , R. Long , K. M. Liew , Y. Xiong , Nat. Commun. 2024, 15, 1273. 38341405 10.1038/s41467-024-45516-4 PMC10858932

[55] C. Lv , K. Huang , Y. Fan , J. Xu , C. Lian , H. Jiang , Y. Zhang , C. Ma , W. Qiao , J. Wang , L. Ling , Nano Energy 2023, 111, 108384.

[56] X.‐F. Qiu , H.‐L. Zhu , J.‐R. Huang , P.‐Q. Liao , X.‐M. Chen , J. Am. Chem. Soc. 2021, 143, 7242. 33956435 10.1021/jacs.1c01466

[57] F. Ma , P. Zhang , X. Zheng , L. Chen , Y. Li , Z. Zhuang , Y. Fan , P. Jiang , H. Zhao , J. Zhang , Y. Dong , Y. Zhu , D. Wang , Y. Wang , Angew.Chem. Int. Ed. 2024, 63, 202412785. 10.1002/anie.202412785 39105415

[58] P. Zhao , H. Jiang , H. Shen , S. Yang , R. Gao , Y. Guo , Q. Zhang , H. Zhang , Angew.Chem. Int. Ed. 2023, 62, 202314121. 10.1002/anie.202314121 37875780

[59] H. Xin , Q. Fu , X. Bao , J. Am. Chem. Soc. 2022, 144, 4874. 35258951 10.1021/jacs.1c12603

[60] R.‐P. Ye , Q. Li , W. Gong , T. Wang , J. J. Razink , L. Lin , Y.‐Y. Qin , Z. Zhou , H. Adidharma , J. Tang , A. G. Russell , M. Fan , Y.‐G. Yao , Appl. Catal. B Environ. 2020, 268, 118474.

[61] X. Wang , H. Shi , J. Szanyi , ACS Catal. 2015, 5, 6337.

[62] F. Wang , S. He , H. Chen , B. Wang , L. Zheng , M. Wei , D. G. Evans , X. Duan , J. Am. Chem. Soc. 2016, 138, 6298. 27135417 10.1021/jacs.6b02762

[63] H. Kang , L. Ma , Y. Liu , Appl. Catal. B Environ. 2024, 352, 124010.

[64] A. Cherevotan , B. Ray , S. R. Churipard , K. Kaur , U. K. Gautam , C. P. Vinod , S. C. Peter , Appl. Catal. B Environ. 2022, 317, 121692.

[65] X. Tang , C. Song , L. Lin , Nat. Commun. 2024, 15, 3115. 38600102 10.1038/s41467-024-47403-4 PMC11006838

[66] S. Kattel , W. Yu , X. Yang , B. Yan , Y. Huang , W. Wan , P. Liu , J. G. Chen , Angew. Chem. Int. Ed. 2016, 55, 7968. 10.1002/anie.201601661 27159088

[67] J. Zhang , S. Deo , M. J. Janik , J. W. Medlin , J. Am. Chem. Soc. 2020, 142, 5184. 32083859 10.1021/jacs.9b12980

[68] Z. Shui , F. Zhang , H. Yang , M. Zhao , Z. Zhao , G. Li , Z. Wei , G. Jiang , Z. Zhang , Z. Hao , Adv. Func. Mat. 2025, 35, 2415774.

[69] Y. Chen , C. Li , J. Zhou , S. Zhang , D. Rao , S. He , M. Wei , D. G. Evans , X. Duan , ACS Catal. 2015, 5, 5756.

[70] K. Kovnir , Y. Grin , J. Am. Chem. Soc. 2010, 132, 14745. 20925320 10.1021/ja106568t

