# Supplementary material for: Niδ+ Atoms Anchored In Situ on Ultrathin Ni‐Phyllosilicate Nanosheet Ensure High‐Efficient CO2 Reduction into CO at Moderate‐Low Temperature
Source: Adv Sci (Weinh). 2025 Nov 27;13(8):e15872. doi: 10.1002/advs.202515872 (PMC12884742; doi:10.1002/advs.202515872)
Supplement: Supplementary file 1 — Supporting Information [file ADVS-13-e15872-s001.docx]

**Support Information**

Ni^δ+^ Atoms Anchored *In-Situ* on Ultrathin Ni-Phyllosilicate Nanosheet Ensure High-Efficient CO_2_ Reduction into CO at Moderate-Low Temperature

Ziluo Ding^+^, Pengfei Li^+^, Qiang Chang^+^, Dongdong Xiao^+^, Xingchen Liu, Wentao Zheng, Hao,Yang, Shan He^*^, Fan Wang^*^, Jianguo Wang^*^, and Fei Wang^*^

Ziluo Ding^+^, Pengfei Li^+^

School of Chemical Science, University of Chinese Academy of Sciences

Beijing 100190 (P. R. China)

Shan He*

School of Light Industry Science and Engineering, Beijing Technology and Business University

Beijing 100048 (P. R. China)

E-mail: [vh30@163.com](mailto:vh30@163.com)

Fan Wang*

Beijing Key Laboratory of Ionic Liquids Clean Process, Institute of Process Engineering, Chinese Academy of Sciences

Beijing 100089 (P. R. China)

E-mail: fwang@ipe.ac.cn

Jianguo Wang*

School of Chemical Engineering, University of Chinese Academy of Sciences

Beijing 100190 (P. R. China)

E-mail: wangjianguo@ucas.ac.cn

Fei Wang*

State Key Laboratory of Coal Conversion, Institute of Coal Chemistry, Chinese Academy of Sciences

Taiyuan 030001 (P. R. China)

E-mail: [wangfei908@sxicc.ac.cn](mailto:wangfei908@sxicc.ac.cn)

Qiang Chang^+^

National Energy Center for Coal to Liquids, Synfuels China Co., Ltd,

Beijing 101400 (P. R. China)

Dongdong Xiao^+^

Beijing National Laboratory for Condensed Matter Physics, Institute of Physics, Chinese Academy of Sciences

Beijing 100190 (P. R. China)

Xingchen Liu

State Key Laboratory of Coal Conversion, Institute of Coal Chemistry, Chinese Academy of Sciences

Taiyuan 030001 (P. R. China)

Wentao Zheng

State Key Laboratory of Coal Conversion, Institute of Coal Chemistry, Chinese Academy of Sciences

Taiyuan 030001 (P. R. China)

Hao,Yang

National Energy Center for Coal to Liquids, Synfuels China Co., Ltd,

Beijing 101400 (P. R. China)

[^+^] These authors contributed equally to this work.

**EXPERIMENTAL SECTION**

**Materials**

Nickel nitrate hexahydrate (Ni (NO_3_)_2_·6H_2_O, 99%) was purchased from Sinopharm group reagent Co. Ltd. sodium carbonate (Na_2_CO_3_, AR) and silicon dioxide (SiO_2_, *S*_BET_=150 m^2^/g) were purchased from Aladdin Biochemical Technology Co. Ltd. Deionized water was used in all the experimental processes.

**Preparation of the catalyst**

The nickel phyllosilicates (Ni−PSNS) catalyst with a Ni loading of 5 wt.% was prepared by deposition-precipitation method. A total of 1.304g Ni (NO_3_)_2_·6H_2_O was dissolved in 225 mL deionized water, followed by the introduction of 5 g of SiO_2_. The temperature was subsequently raised to 70 °C and the mixture was stirred for 30 min. The pH of the suspension was adjusted to 9 by slowly dropwise adding a 0.01 M Na_2_CO_3_ aqueous solution and aged at 70 °C for 1h under continuous stirring. Then the suspension was centrifuged and washed by deionized water for three times and subsequently dried overnight at 120 °C. The resulting dried catalyst is referred to as Ni−PSNS. The Ni−PSNS sample was directly *in situ* reduced by 10% H_2_/N_2_ for 2 hours at 400 °C without a calcination process, using a heating rate of 5 °C/min. This resulted in the formation of abundant Ni*^δ+^* atoms with low electron density anchored *in situ* on ultrathin Ni-phyllosilicate nanosheet, which were designated as a-Ni*^δ+^*−PSNS. The Ni/NiSiO*_x_* sample was synthesized by *in situ* reducing Ni−PSNS sample at 700 °C for 2 hours.

**Catalyst characterization**

The X-ray diffraction (XRD) patterns of samples were acquired on a Bruker D2 Advance powder diffractometer equipped with Cu-Kα radiation (λ = 1.54178 Å), operating at 30 kV and 10 mA. The JEOL ARM200F aberration-corrected scanning transmission electron microscope, equipped with dual energy-dispersive X-ray spectroscopy (EDS) detectors, was employed to conduct atomic-resolution high-angle annular dark-field (HAADF-STEM) imaging and corresponding elemental mapping. X-ray photoelectron spectroscopy (XPS) analysis without exposure to air was conducted using a Thermo Scientific K-Alpha spectrometer equipped with a source of Al Kα. All binding energies were referenced to the adventitious carbon C 1s peak at 284.6 eV to account for surface charging effects. Prior to analysis, the sample underwent *in situ* reduction under flowing pure 10% H_2_/N_2_ for 2 hours, followed by cooling to room temperature (RT). The pretreated sample was then transferred directly to the analysis chamber under inert conditions to prevent air exposure. The X-ray absorption near edge structure (XANES) spectroscopy was performed at SSRF BL17B1 of the National Facility for Protein Science in Shanghai (NFPS), Shanghai Advanced Research Institute, Chinese Academy of Sciences (CAS). Spectra were collected in the fluorescent mode with a Lytle detector. The corresponding XANES analysis was performed using Athena and Artemis software.

The H_2_-temperature-programmed reaction (H_2_-TPR) profiles and temperature programmed desorption (TPD) profiles were acquired using a Micromeritics ChemiSorb 2920 equipped with a thermal conductivity detector (TCD) and coupled with an online mass spectrometer. For H_2_-TPR, 200 mg sample was loaded into a quartz U-tube reactor and pretreated under flowing argon at 150 °C for 1 hours to eliminate adsorbed moisture. The reduction profile was subsequently recorded in a H_2_/Ar mixed gas stream (1:9 v/v ratio, total flow rate 50 mL·min^-1^) using a controlled heating ramp of 10 °C·min^-1^ from 50 °C to 800 °C. For CO-TPD, 1 g sample was first reduced under flowing pure 10% H_2_/N_2_ (50 mL·min^-1^) at different reduction temperature (400 °C and 700 °C) respectively for 2 hours. The reduced sample was then purged with argon (Ar) for 30 minutes to remove residual hydrogen, followed by cooling to 50°C to facilitate 5% CO-He adsorption for 30 min. As the baseline was stable, TPD measurements were conducted from 50 °C to 800 °C under helium at a heating rate of 10 °C·min^-1^. For the H_2_-TPD process, 200 mg sample was loaded into a U-tube, reduced under 10% H_2_/N_2_ flow (50 mL·min^-1^) at different reduction temperature (400 °C and 700 °C) respectively for 2 hours, and then cooled to 50 °C. Prior to temperature-programmed desorption (TPD) analysis, the system was purged with argon (Ar, 50 mL·min^-1^) for 30 minutes to remove residual H_2_. TPD measurements were subsequently conducted with a heating rate of 10 °C·min^-1^ up to 800 °C. H-D exchange experiment was performed using tubular furnace with an online mass spectrometer. 200 mg sample was reduced at 400 and 700 °C *in situ* respectively in a 10% H_2_/N_2_ flow (50 mL/min) for 2h and then cooled to 50 °C. Subsequently, the reduced sample was purged with H_2_/D_2_/N_2_ (5:5:90 v/v ratio, total flow rate 50 mL·min^-1^) until the baseline was stable. H-D exchange measurements were conducted from 50 °C to 600 °C under H_2_/D_2_/N_2_ at a heating rate of 10 °C·min^-1^. For the CO+H_2_-TPSR, 200 mg sample was reduced at 400 and 700 °C *in situ* respectively in a 10% H_2_/N_2_ flow (50 mL/min) for 2h and then cooled to 50 °C. After that, CO/H_2_/He (45:54:10 v/v ratio, total flow rate 20 mL·min^-1^) was introduced until the baseline was stable. Subsequently, the TPSR measurement was performed from 50 to 400 °C with a temperature ramp of 10 °C/min. The dispersion of Ni metal particles on the catalysts was assessed using the H_2_ chemisorption technique employing a Micromeritics Auto Chem II 2920 apparatus equipped with a thermal conductive detector. In each experiment, a sample weighing 300 mg was subjected to reduction at a specified temperature with a hydrogen flow rate of 50 mL/min for 2 h, followed by cooling to 40 °C under an Ar flow. The nickel dispersion and Surface Ni^0^ concentration were calculated based on an assumed Ni0/H adsorption stoichiometry factor of 2.

Infrared spectroscopy was performed using a Vertex70 (Bruker) spectrophotometer with a resolution of 4 cm^−1^. *In situ* IR experiment was performed over Bruker infrared spectrometer with the transmission cell equipped with a high-temperature reaction chamber. For the variation of -OH, the Ni−PSNS sample (30 mg) were pressed into self-supporting wafers with a diameter of 12 mm. Prior to measurement, the Ni−PSNS sample was pretreated under evacuated conditions at 200 °C for 30 min, whose spectra were taken as the background spectra. After that, the Ni−PSNS sample was *in situ* reduced under flowing pure 10% H_2_/Ar at 400 °C and 700 °C respectively for 2 hours, followed treated under vacuum conditions for another 30 min before cooling to 200 °C. The spectra variation process of -OH was measured at 200 °C under vacuum condition. When the infrared spectrometer is in transmission mode, the -OH peak of the Ni−PSNS appears as a negative peak. Since the Ni−PSNS sample is used as the background reference, the -OH peaks of a-Ni*^δ+^*−PSNS and Ni/NiSiO*_x_* exhibit positive peaks due to -OH removal caused by H_2_ *in situ* reduction. The intensity of these positive peaks is proportional to the amount of -OH groups eliminated.

*In situ* diffuse reflectance infrared Fourier transform spectroscopy (DRIFTS) measurements were conducted using a Bruker Vertex70 spectrometer equipped with a high-temperature reaction chamber and a mercury cadmium telluride (MCT) detector, with spectra collected at a resolution of 4 cm^-1^. Before the test, 50 mg of catalyst was pretreated in 10% H_2_/N_2_ at different reduction temperature (400 °C and 700 °C) for 2 hours and then purged with He at 400 °C for 30 minutes to remove residual hydrogen, followed by cooling to 300 °C, whose stable spectra were taken as the background spectra. For *in situ* time-resolved DRIFTS spectra under working conditions, 20 % CO_2_/N_2_ (45 mL min⁻¹) was injected into the reaction cell for 30 min and the temporal evolution of the IR spectrum was recorded. Subsequently, the H_2_ was injected in the presence of CO_2_ with a mixed gas of 45% CO_2_/45% H_2_/N_2_ at a total flow rate of 20 mL min^-1^for 30 min and the evolution was again observed. For the conversion path of formate intermediates identified by *in situ* DRIFTS spectra with programmed heating, the CO_2_ + H_2_/N_2_ mixed gas was first injected into the reaction cell of *in situ* DRIFTS spectroscopy at 200 °C; after CO_2_ was completely consumed and converted to formate species, the system temperature rose from 200 °C to 300 °C under H_2_/N_2_ to observe the conversion of formate species, whose spectra were recorded regularly. Similarly, the reactant gas (45% CO_2_/45% H_2_/10% N_2_) at a flow rate of 20 mL min^-1^ was transferred into the reaction cell at desired temperatures (200 ~ 300 °C) and spectra were recorded. The conversion process of formate intermediates in a tube furnace coupled with a MASS spectrometer to further validate catalytic mechanism of a-Ni^δ+^−PSNS(400). Before the test, 50 mg of catalyst was pretreated in 10% H_2_/N_2_ at 400 °C for 2 hours and then purged with He at 400 °C for 30 minutes to remove residual hydrogen, followed by cooling to 200 °C. Subsequently, the CO_2_ + H_2_ mixed gas was first injected at 200 °C for 30min until the baseline was stable. Then the system temperature rose from 200 °C to 300 °C under H_2_ to observe the conversion of formate species, whose MASS spectra were recorded regularly.

For the CO-DRIFTS, the catalyst was initially purged with pure He at a flow rate of 20 mL min^-1^ under ambient temperature (RT). The spectra collected during this step served as the background reference. Subsequently, 5% CO/He gas mixture (flow rate: 20 mL min^-1^) was introduced into the reaction cell. The system was equilibrated for 30 minutes to achieve saturation adsorption, during which the corresponding spectra were recorded. Following CO exposure, the catalyst was purged with pure He (20 mL min^-1^) and Spectra were continuously monitored throughout the He purge to track desorption dynamics.

**Evaluation of the catalytic performance**

The catalytic performance of the synthesized catalysts was evaluated in a fixed-bed flow reactor with a tubular stainless-steel tube (16 mm internal diameter). Prior to the catalytic performance test, 300 mg of catalyst was pretreated in 10% H_2_/N_2_ at 400 °C for 2h, and then cooled to room temperature. The reaction gas consisting of 45% CO_2_ and 45% H_2_ (CO_2_:H_2_=1:1) balanced with N_2_ was fed at a flow rate of 50 mL min^-1^ (WHSV=10000 mL g_cat_^-1^ h^-1^). The sample was heated to temperature range from 220-300 °C and then kept for 2h until the catalytic reaction reached a steady state. The products were analyzed online by gas chromatograph (Agilent GC 7900) equipped with a flame ionization detector (FID) and a thermal conductivity detector (TCD). CO_2_, CO and internal standard N_2_ were analyzed with TCD, while CH_4_ and other hydrocarbons were analyzed with FID. The conversion of CO_2_, selectivity of CH_4_ and CO and space-time yield (STY) were calculated as follows:

$$\text{CO}_{2} \text{conversion} \text{(\%)=}\frac{\frac{\text{A}_{\text{CO}_{\text{2in}}}}{\text{A}_{\text{N}_{\text{2in}}}}\text{-}\frac{\text{A}_{\text{CO}_{\text{2out}}}}{\text{A}_{\text{N}_{\text{2out}}}}}{\frac{\text{A}_{\text{CO}_{\text{2in}}}}{\text{A}_{\text{N}_{\text{2in}}}}}$$

$$\text{CO} \text{selectivity} \text{(\%)=}\frac{\text{n}_{\text{CO}}}{\text{n}_{CO_{2\text{in}}}-\text{n}_{CO_{2\text{out}}}}$$

$$\text{STY}_{\text{CO}}\left(\text{mmol}_{\text{CO}} \text{g}_{\text{cat}}^{\text{-1}} \text{h}^{\text{-1}}\right)\text{ =}\frac{V_{CO_{2}}\times X_{_{CO_{2}}}\times S_{_{\text{CO }}}}{m_{cat}\times Mv}$$

Where A_CO2in_ and A_CO2out_ represent peak areas of CO_2_ in the feed and effluent. X_CO2in_ and S_CO_ represent the conversion of CO_2_ and selectivity of CO.

The calculation formulas for the exposed quantity of Ni^δ+^ atoms of a-Ni^δ+^−PSNS(300) and a-Ni^δ+^−PSNS(400) and the TOF value of a-Ni^δ+^−PSNS(400) based on *semi-in situ* Ni 2p_3/2_ XPS spectra (Figure S9) were presented below:

$$TOF=\frac{\text{the genated number of CO (or CH}_{4}\text{) per second}}{\text{the number of active sites}}$$

$$\text{N}_{\text{active sites of catalysts}}=\frac{\text{Ni wt\%}\times R}{M_{N\text{i}}}$$

$$R=\frac{Ni^{\delta+}}{Ni^{\delta+}+Ni^{2+}}=\frac{\text{ }integral\text{ }area\text{ }of\text{ }Ni^{\delta+}}{integral\text{ }area\text{ }of\text{ }Nispecials}$$

Where integral area of Ni^δ+^ and Ni (Ni^δ+^+Ni^2+^) was obtained from semi-in situ Ni 2p_3/2_ XPS (Figure S9).

$$T\text{he genated number of CO (or CH}_{4}\text{) per second=}\frac{V_{CO_{2}}\times X_{_{CO_{2}}}\times S_{_{\text{CO }}}(or\text{ }S_{CH_{4}})}{m_{cat}\times Mv}$$

$$TOF=\frac{\text{the genated number of CO (or CH}_{4}\text{) per second}}{\text{the number of active sites}}=\frac{X_{CO_{2}}\times S_{CO}(or\text{ }S_{CH_{4}})\times V_{CO_{2}}\times M_{Ni}}{m_{cat}\times\text{Ni wt\%}\times R\times Mv}$$

Where m_cat_ was weight of catalysts in reaction, Ni species wt% was the loading of Ni in total catalyst, V_CO2_ was the flow rate of CO_2_ (mL/s), *Mv* was the molar volume of the ideal gas (22400 mol/L), M_Ni_ was the molecular weight of Ni.

The calculation formulas for the exposed quantity of Ni^0^ sites of Ni/NiSiO_x_(500), Ni/NiSiO_x_(600), and Ni/NiSiO_x_(700) and the TOF value of Ni/NiSiO_x_(700) based on H_2_-pulse adsorption were also presented below:

$$\text{N}_{\text{active sites of catalysts}}=\frac{2\times V_{\text{quantitative loop}}\times S_{\text{adsorption}}}{Mv\times S_{\text{standard pulse peak}}\times m_{cat}}$$

$$T\text{he genated number of CO (or CH}_{4}\text{) per second=}\frac{V_{CO_{2}}\times X_{_{CO_{2}}}\times S_{_{\text{CO }}}(or\text{ }S_{CH_{4}})}{Mv\times m_{cat}}$$

$$TOF=\frac{\text{the genated number of CO (or CH}_{4}\text{) per second}}{\text{the number of active sites}}$$

$$D=\frac{\text{N}_{\text{active sites of catalysts}}\times M_{Ni}}{Ni\text{ }wt\%}$$

Where V_quantitative loop_ was the volume of quantitative loop, S_adsorption_ was the area of the H_2_ adsoption peak and *Mv* was the molar volume of the ideal gas. $S_{\text{standard pulse peak}}$was the area of the standard pulse peak. D was the metal dispersion (%).

**DFT calculations**

**Methods**

All calculations were performed using the plane-wave-based periodic DFT method implemented in the Vienna ab initio simulation package (VASP),^[1-2]^ where the ionic cores are described by the projector augmented wave (PAW) method.^[3-4]^ The exchange and correlation energies are computed using the Perdew-Burke-Ernzerhof functional(PBE).^[5]^ The van der Waals interactions were considered using the DFT-D3 semiempirical method.^[6]^ To achieve accurate energies with errors of less than 1 meV per atom, the cutoff energy was set at 400 eV. The Gaussian electron smearing method with σ = 0.1 eV and ISMEAR = 0 were used. Geometric optimization converged until the forces acting on the atoms were smaller than 0.02 eV·Å^−1^, whereas the energy threshold-defining self-consistency of the electron density was set to 10^−5^ eV. Spin polarization was included to correctly describe magnetic properties, which is essential for an accurate description of all energetic data All transition state structures were optimized by using the climbing image nudged elastic band (CI-NEB) method,^[7]^ and the frequency analysis was also processed to verify an authentic transition state having only one imaginary frequency.

**Models**

The models utilized in our theoretical calculations were based on the experimental characterization results (XRD and TEM, Figure 1 and 2, respectively). Si_2_Ni_3_H_4_O_9_ was utilized to represent our Ni−PSNS catalyst for theoretical calculations. The (001) surface of different models was employed in the whole calculation process owing to the (001) crystal planes are the main exposed crystal planes. The Si_2_Ni_3_H_4_O_9_ (001) surface was cleaved from the Si_2_Ni_3_H_4_O_9_ unit cell (a = b = 5.288 Å, c = 7.209 Å, Figure S28) crystal structure containing 18 atoms with space group P31m (Material ID: MP-1105877). A Si_2_Ni_3_H_4_O_9_ two layers periodic (2 × 2) supercell surface model (i.e., Ni−PSNS model used in theoretical calculations) was constructed with all atoms fully relaxed during optimization (Figure S29). a-Ni*^δ+^*−PSNS model was obtained by removing three outermost hydroxyl groups on the perfect Si_2_Ni_3_H_4_O_9_(001) surface of Ni−PSNS model (Figure S30).

Since SiO_2_, as support, exhibits no catalytic activity in our study, the Ni(111) surface was applied in calculations as the model to represent the Ni/NiSiO*_x_* catalyst. The Ni(111) surface was cleaved from the Ni unit cell (a = b = c = 3.524 Å, Figure S31) crystal structure with 4 atoms from the database of the Material Studio. A Ni four layers periodic (4 × 4) supercell surface model was constructed with all atoms fully relaxed during optimization (Figure S32). A (3 × 3 × 1) k-point grid was utilized for sampling the Brillouin zone, and a 15 Å vacuum was introduced between the repeated slabs along the z-direction to avoid significant interactions. The similar surfaces had also been reported in previous work with the same models and methods as we utilized in our work.^[8-9]^

The reaction barrier (*E*_a_) and reaction energy (*E*_r_) are calculated according to *E*_a_ = *E*_TS_ – *E*_IS_ and *E*_r_ = *E*_FS_ – *E*_IS_, where *E*_IS_, *E*_FS_ and *E*_TS_ are the energies of the corresponding initial state (IS), final state (FS), and transition state (TS), respectively. All reported energetic data included zero-point energy (ZPE) correction through processing the frequency analysis by vaspkit.1.3.3.^[10]^


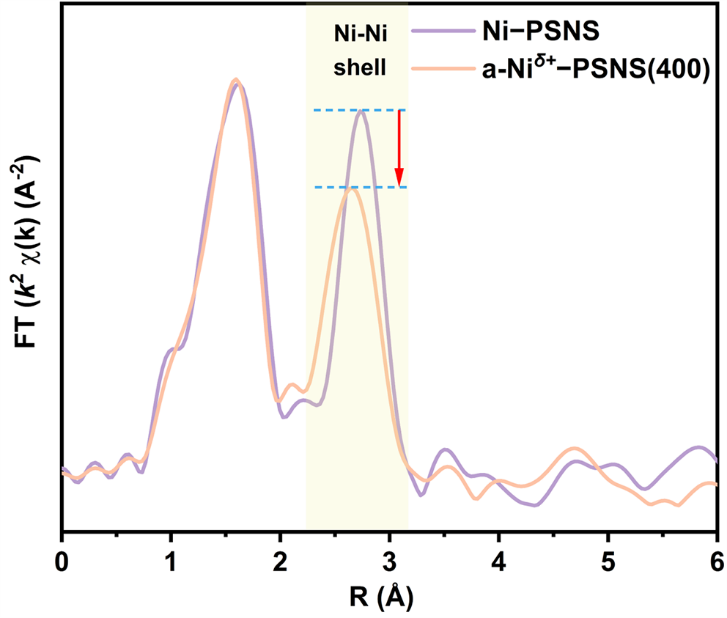


**Figure S1.** Fourier-transformed *k*^2^-weighted EXAFS spectra at Ni *K*-edge for Ni−PSNS and a-Ni*^δ+^*−PSNS(400)


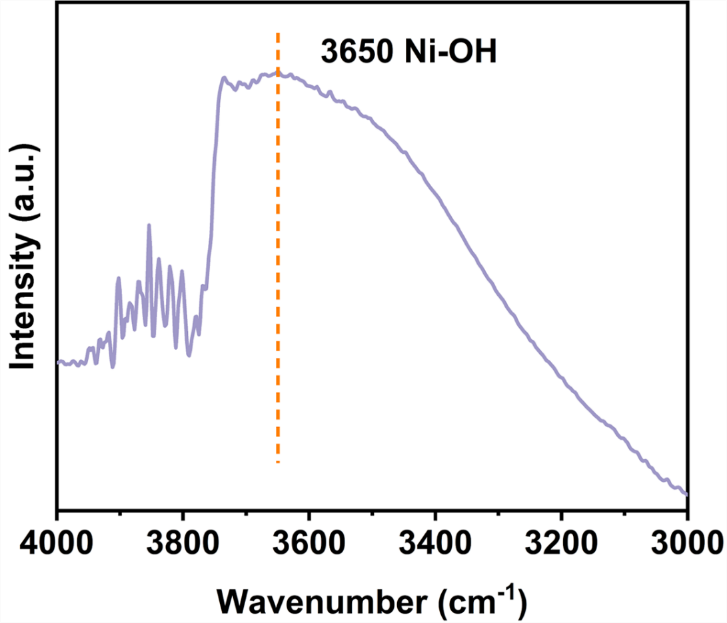


**Figure S2.** *In situ* FT-IR spectrum of Ni−PSNS from 3000 to 4000 cm^-1^


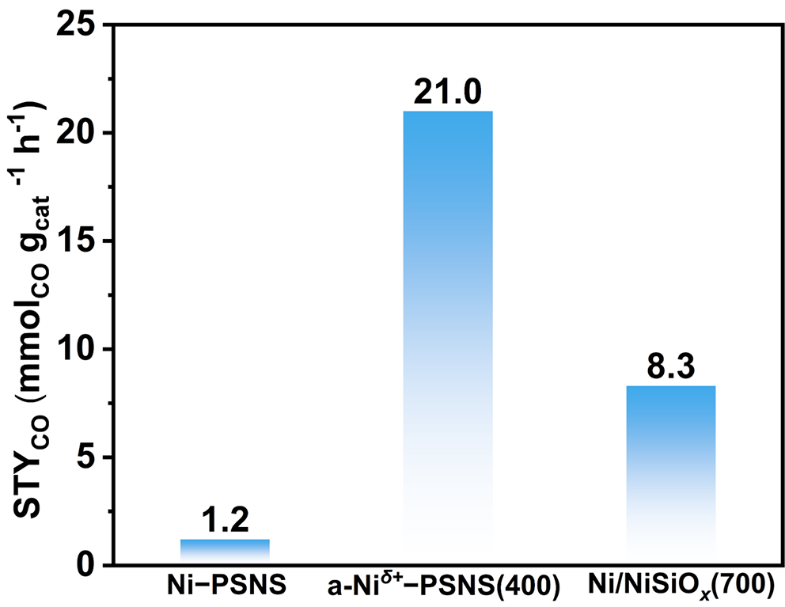


**Figure S3.** CO formation rates at 300°C of Ni−PSNS, a-Ni*^δ+^*−PSNS(400) and Ni/NiSiO*_x_*(700).


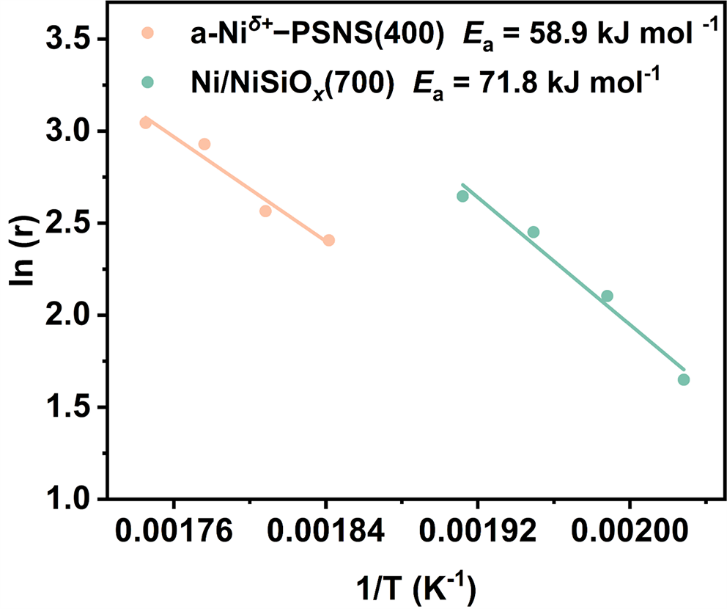


**Figure S4.** Arrhenius plots and the apparent activation energy (*E*_a_) for the catalysts of a-Ni*^δ+^*−PSNS(400) and Ni/NiSiO*_x_*(700).


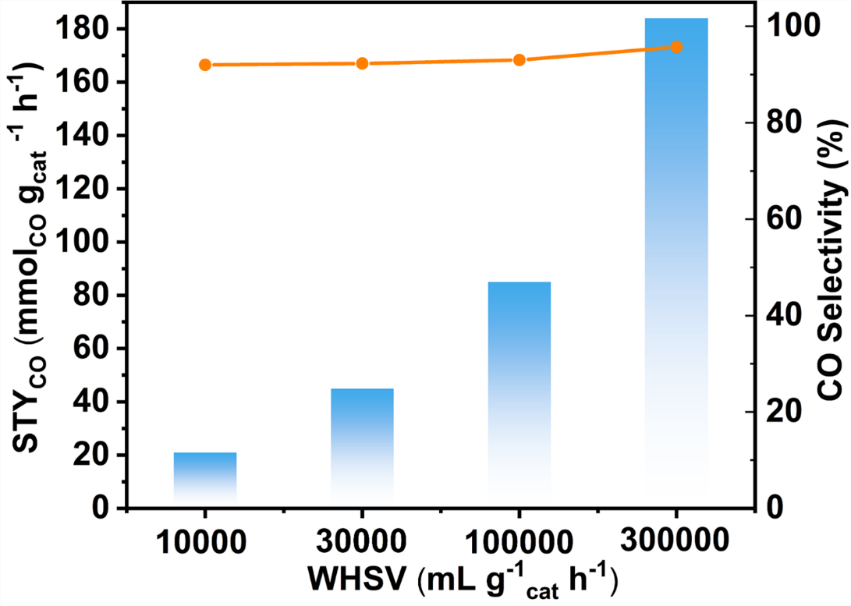


**Figure S5.** The catalytic performance of a-Ni*^δ+^*−PSNS(400) at 300 °C with various WHSV from 10000 to 300000 mL g_catal_^−1^ h^−1^.

**The structural characterizations, catalytic performances, and results and discussion of various Ni-based catalysts**


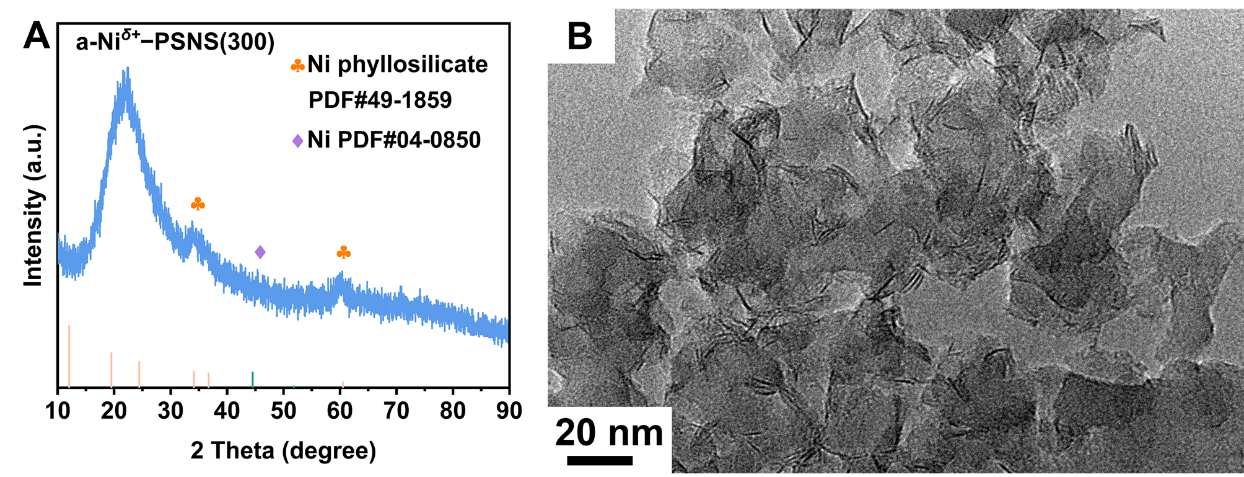


**Figure S6.** (A) *Semi-in situ* XRD patterns of a-Ni^δ+^−PSNS(300). (B) TEM images of a-Ni^δ+^−PSNS(300).


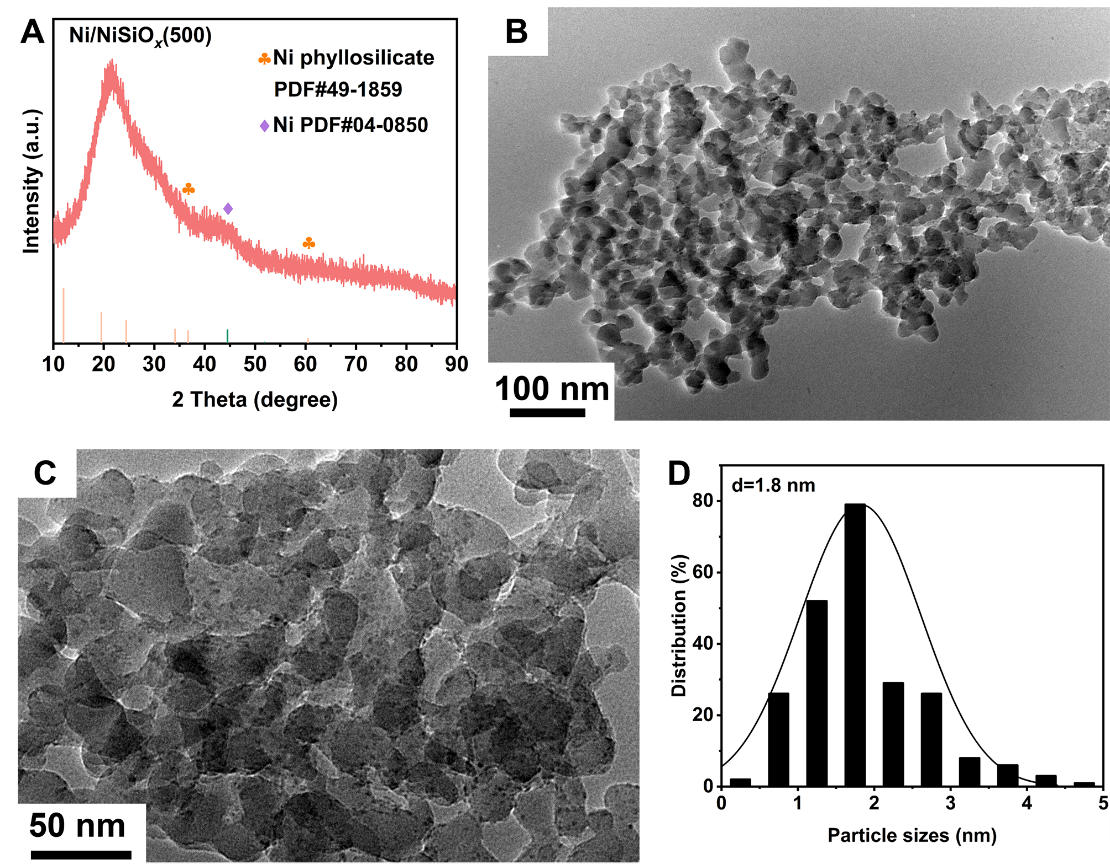


**Figure S7.** (A) *Semi-in situ* XRD patterns of Ni/NiSiO*_x_*(500). TEM images of Ni/NiSiO*_x_*(500) at (B) low magnification and (C) high magnification. (D) Histogram of size distribution of Ni nanoparticles dispersed on Ni/NiSiO*_x_*(500).


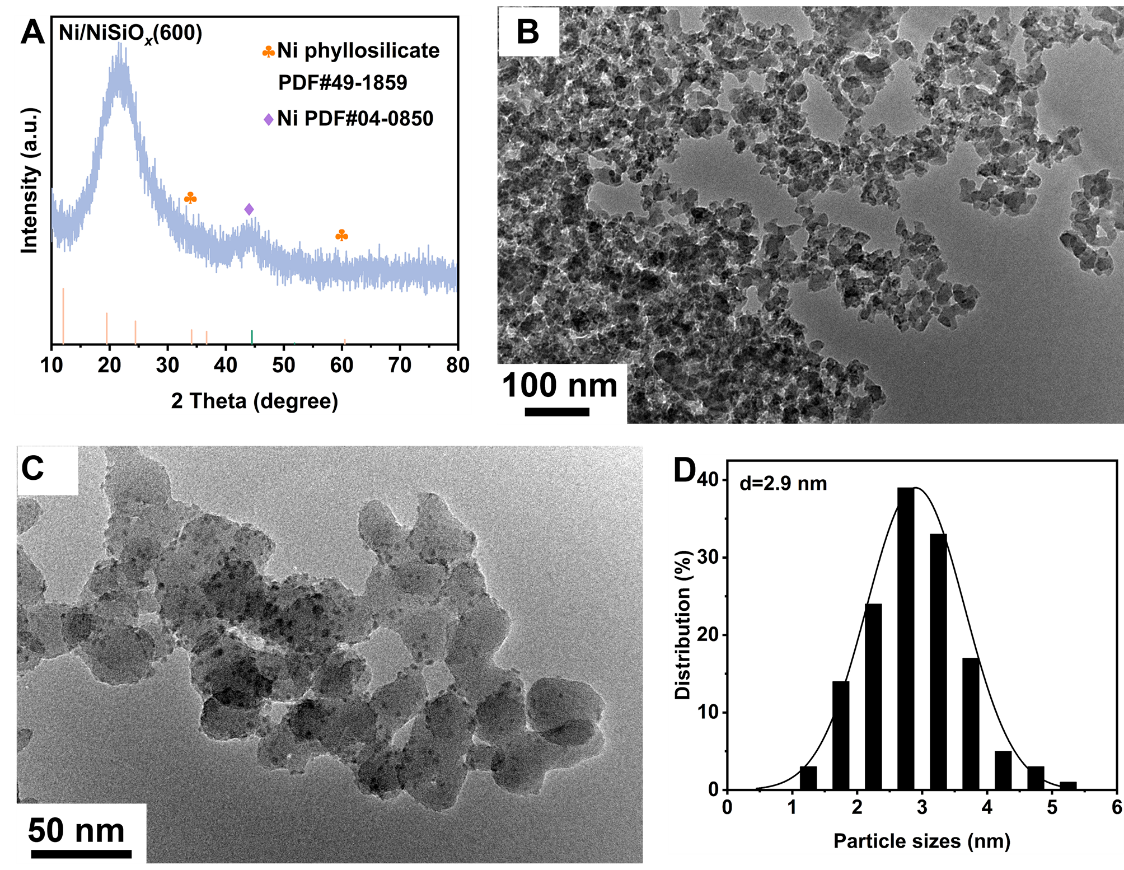


**Figure S8.** (A**)** *Semi-in situ* XRD patterns of Ni/NiSiO*_x_*(600). TEM images of Ni/NiSiO*_x_*(600) at (B) low magnification and (C) high magnification. (D) Histogram of size distribution of Ni nanoparticles dispersed on Ni/NiSiO*_x_*(600).


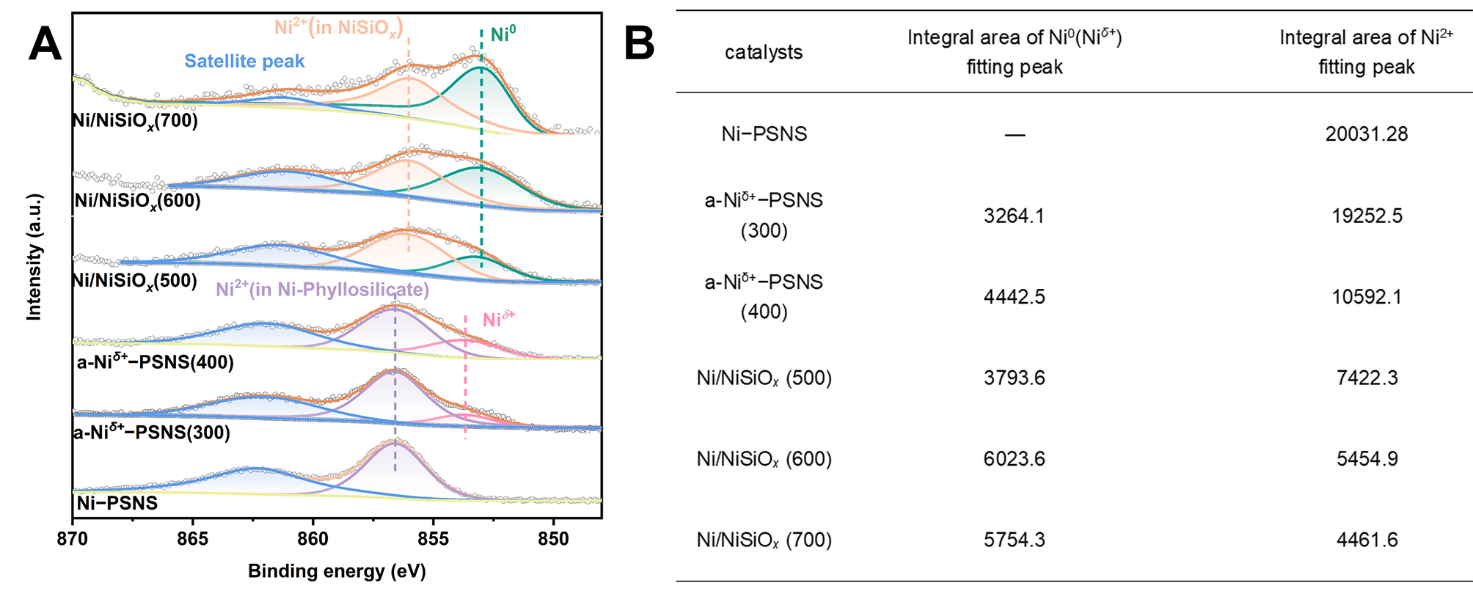


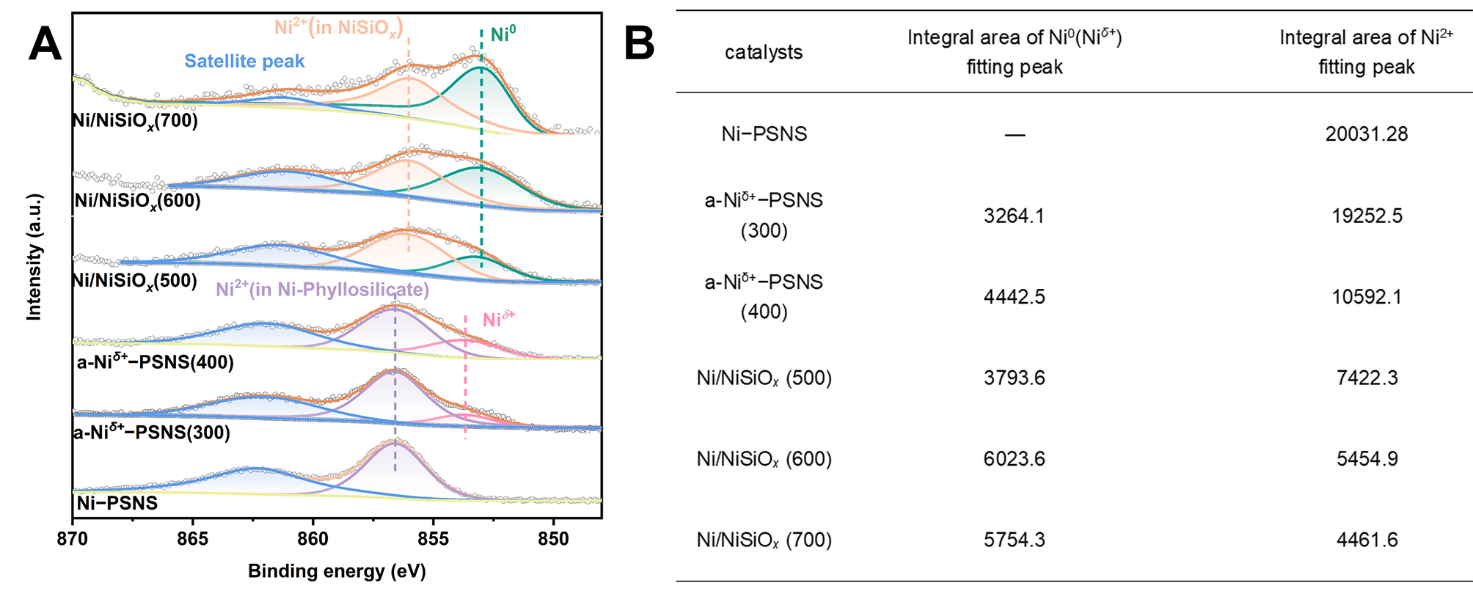


**Figure S9.** (A) *Semi-in situ* XPS Ni 2p_3/2_ spectrum of various Ni-based catalysts. (B) Integral area of fitting peaks attributed to Ni*^2+^*, Ni*^δ+^*, and Ni^0^.


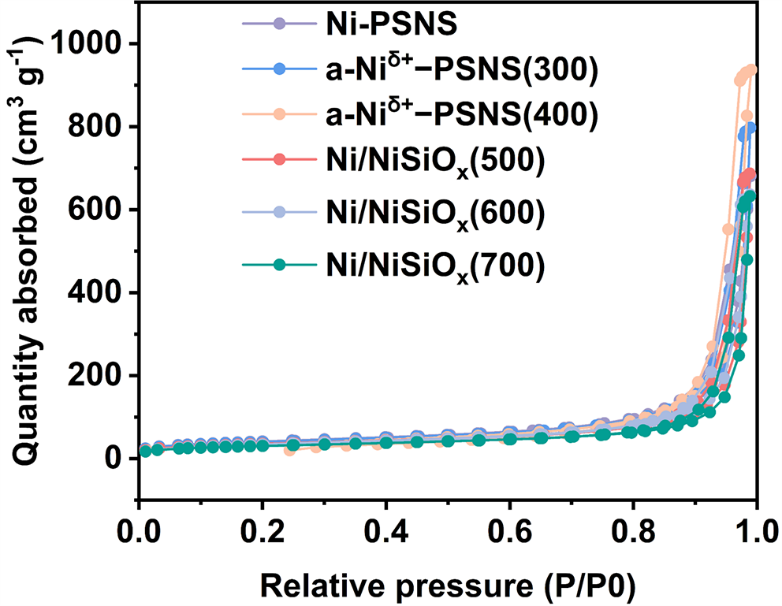


**Figure S10.** N_2_ adsorption-desorption isotherms of various Ni-based catalysts.

We have prepared various Ni-based catalysts under *in situ* reduction temperature from 300 °C to 700 °C respectively, obtaining a-Ni*^δ+^*−PSNS(300), a-Ni*^δ+^*−PSNS(400), Ni/NiSiO*_x_*(500), Ni/NiSiO*_x_*(600), and Ni/NiSiO*_x_*(700). From various structural characterization results (Figure 1−3, Figure S6−S10, and Table S2), as the *in situ* reduction temperature of the ultrathin Ni-phyllosilicate increased, the resulting series of catalysts undergone significant structural evolution. Under *in situ* reduction temperatures of 300 °C and 400 °C, both the obtained a-Ni*^δ+^*−PSNS(300) and a-Ni*^δ+^*−PSNS(400) showed Ni*^δ+^* atoms with low electron density anchored *in situ* on ultrathin Ni-phyllosilicate nanosheet without obvious Ni^0^ species. Meanwhile, as the reduction temperature increased from 300 °C to 400 °C, the amount of Ni*^δ+^* atoms also increased significantly. When the *in situ* reduction temperature increased to 500 °C, the structure of ultrathin Ni-phyllosilicate nanosheet disappeared, accompanied by the appearance of NiSiO*_x_*-supported Ni^0^ nanoparticles. Correspondingly, a series of supported Ni-based catalysts, including Ni/NiSiO*_x_*(500), Ni/NiSiO*_x_*(600), and Ni/NiSiO*_x_*(700), were obtained when the *in suit* reduction temperature was raised from 500 to 700 °C. Moreover, as the *in suit* reduction temperature increased from 500 °C, the particle size of the resulting Ni^0^ nanoparticles increased, while the dispersion of Ni^0^ sites decreased.


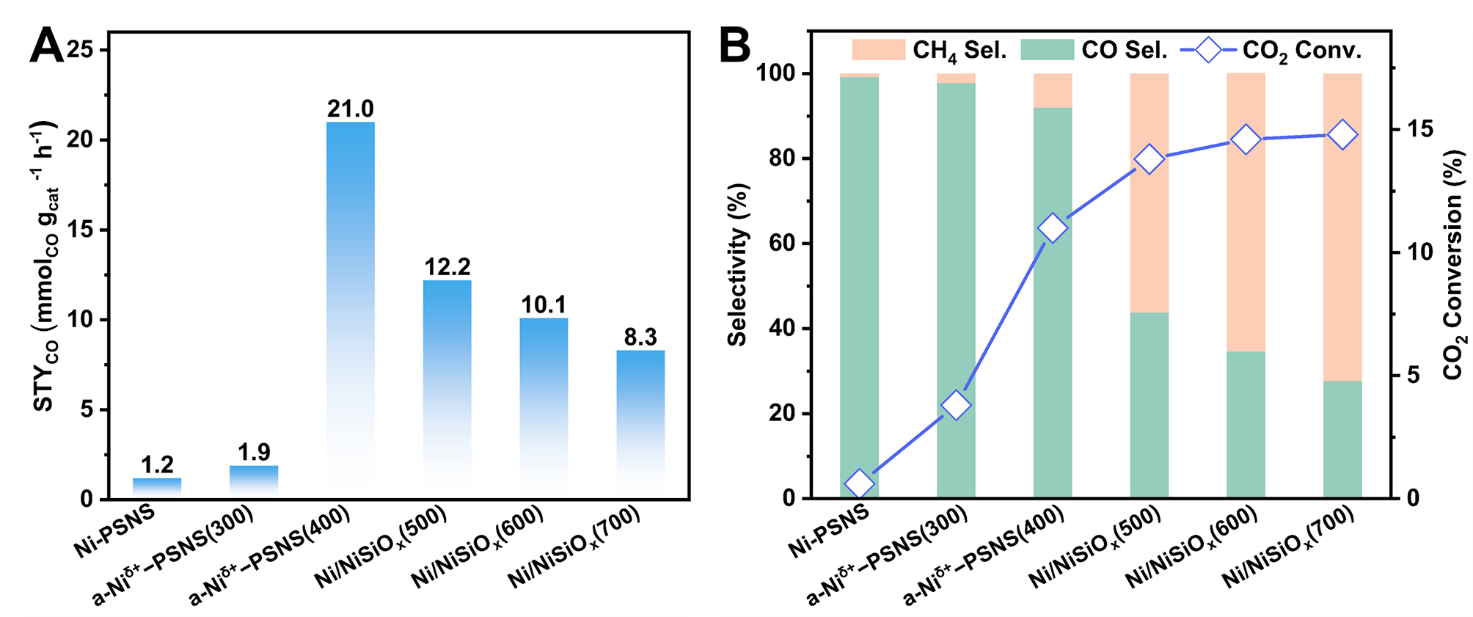


**Figure S11.** (A) CO formation rate of various Ni-based catalysts. (B) CO_2_ conversion and selectivity of various Ni-based catalysts. MLT-RWGS reaction conditions: 0.3 g of catalyst, 45:45:10 CO_2_/H_2_/N_2_, and WHSV= 10000 mL g_catal_^−1^·h^−1^ at 300 °C.

The trend of catalytic performance changes of these catalysts can be well matched with their structural evolution mentioned above (Figure 1−3, Figure S6−S10, Figure S11, and Table S2). It was found that as the *in situ* reduction temperature increased from 300 to 700 °C, the CO formation rate of the obtained catalyst changed in a volcanic manner, with a maximum CO formation rate present in a-Ni*^δ+^*−PSNS(400). This can be reasonably explained as: as the reduction temperature increased from 300 °C to 400 °C, the amount of Ni*^δ+^* atoms also increased significantly without producing Ni^0^ species, endowing a-Ni*^δ+^*−PSNS(400) with more active sites favorable for MLT-RWGS and resulting in higher CO formation rate; and compared with a-Ni*^δ+^*−PSNS(300), the CO selectivity of a-Ni*^δ+^*−PSNS(400) slightly decreased, which may be due to that the increase of Ni*^δ+^* atoms promoted CO_2_ hydrogenation to CH_4_ to some extent; when the *in situ* reduction temperature continued to rise to 500 °C, the obtained Ni/NiSiO*_x_*(500) with high-dispersion and small particle size of Ni^0^ nanoparticles promoted significantly the side reaction of methanation, thus leading to a sharp decrease in CO formation rate; moreover, as the reduction temperature increased from 500 to 700 °C, the methanation of the obtained supported Ni-based catalyst was intensified, accompanied by a gradual decrease in CO formation rate, i.e., the CO formation rate of the three supported Ni nanoparticle catalysts during MLT-RWGS that decrease in the following order: Ni/NiSiO*_x_*(500) (12.2 mmol_CO_ h^−1^ g_cat_^−1^) > Ni/NiSiO*_x_*(600) (10.1 mmol_CO_ h^−1^ g_cat_^−1^) > Ni/NiSiO*_x_*(700) (8.3 mmol_CO_ h^−1^ g_cat_^−1^); this result was attributed to that the Ni^0^ nanoparticles with larger particle size and lower dispersibility could lead to higher catalytic activity for methanation, which was also consistent with previous research reports.^[11]^

**The structural characterizations, catalytic performances, and results and discussion of a-Ni^δ+^-PSNS(400-15) with about Ni loading of 15%.**

**
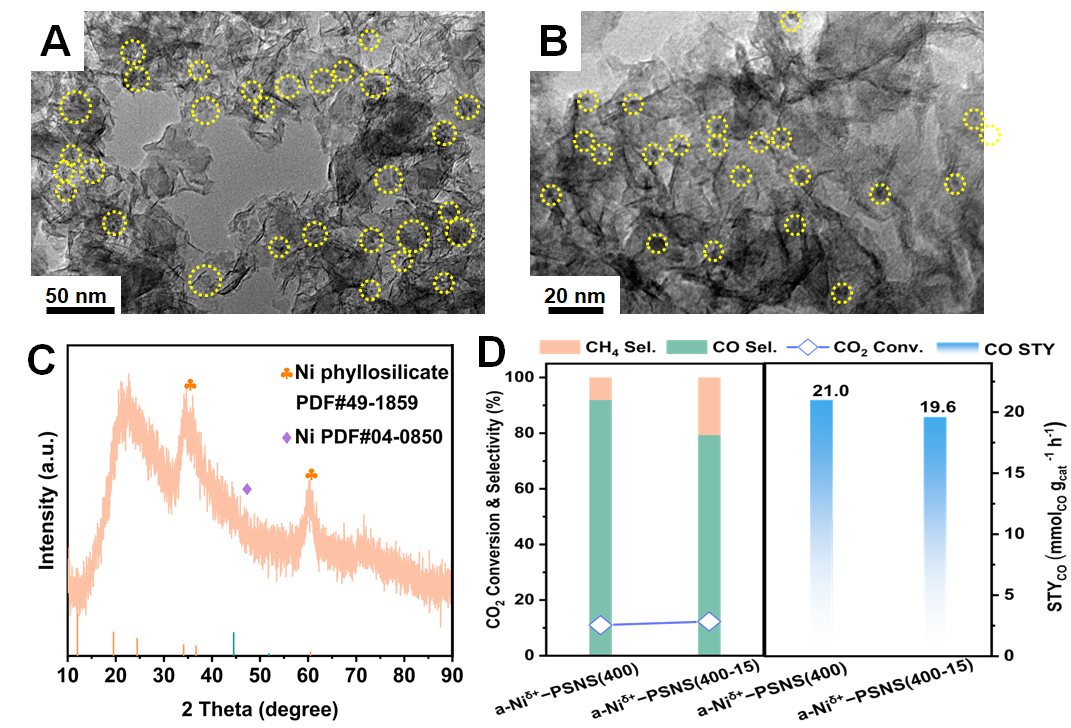
**

**Figure S12.** TEM images of a-Ni^δ+^-PSNS(400-15) at (A) low magnification and (B) high magnification. (C) *Semi-in situ* XRD patterns of a-Ni^δ+^-PSNS(400-15). (D) CO_2_ conversion, CO selectivity, and CO formation rate of a-Ni^δ+^-PSNS(400) and a-Ni^δ+^-PSNS(400-15)**.** MLT-RWGS reaction conditions: 0.3 g of catalyst, 45:45:10 CO_2_/H_2_/N_2_, and WHSV= 10000 mL g_catal_^−1^·h^−1^ at 300 °C.

We have prepared a-Ni^δ+^-PSNS(400-15) catalyst with about Ni loading of 15%. TEM images of a-Ni^δ+^-PSNS(400-15) displayed typical ultrathin Ni phyllosilicate nanosheet, while also observing a small amount of tiny Ni nanoparticles highly dispersed on the nanosheets (Figure S12A−B). In addition, XRD of a-Ni^δ+^-PSNS(400-15) showed characteristic diffraction peaks of 1:1 type Ni phyllosilicate, but no obvious diffraction peaks attributed to metallic Ni^0^ phase were observed, which may be attributed to the small amount, tiny particle size, and high dispersion of Ni^0^ nanoparticles present in a-Ni^δ+^-PSNS(400-15) (Figure S12C). This result was also supported by observations from TEM. Next, from the catalytic evaluation results for (MLT-RWGS) (Figure S12D), the CO_2_ conversion of a-Ni^δ+^-PSNS(400-15) increased while its CO selectivity decreased compared to a-Ni^δ+^-PSNS(400) (12.3% vs. 11.0% for CO_2_ conversion and 79.4% vs. 92.0% for CO selectivity), and correspondingly methanation was further driven, resulting in a slight decrease in CO formation rate (19.6 mmol_CO_ h^−1^ g_cat_^−1^). This was likely due to a small amount of tiny Ni nanoparticles present in a-Ni^δ+^-PSNS(400-15), as revealed in the results of TEM and XRD, promoted methanation and thus reduced CO selectivity and CO formation rate.


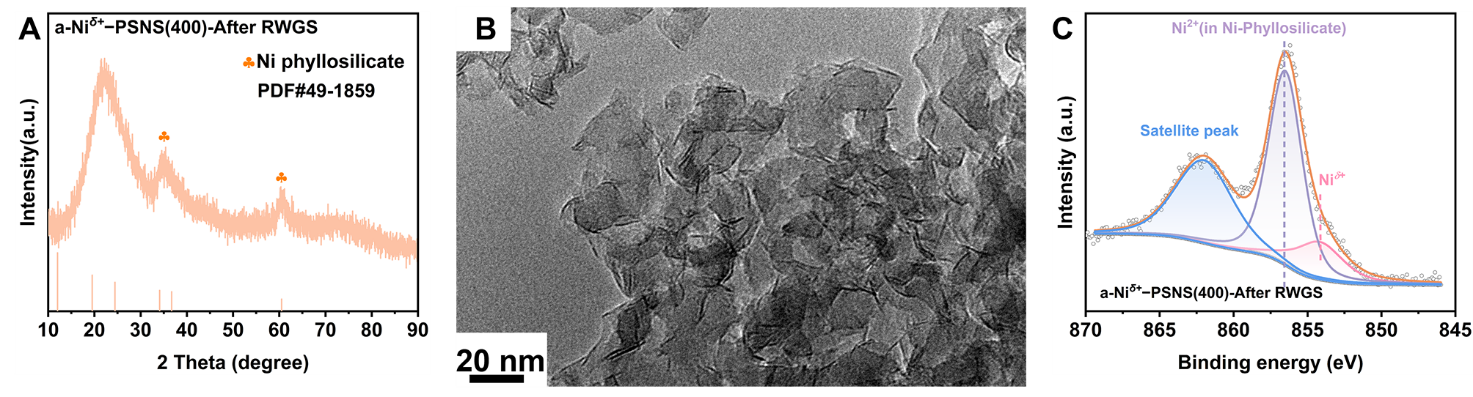


**Figure S13.***Semi-in situ* XRD patterns (A), TEM images (B), and *semi-in situ* XPS Ni 2p_3/2_ spectrum (C) of a-Ni^δ+^-PSNS(400) after the long-term stability of 150 h.


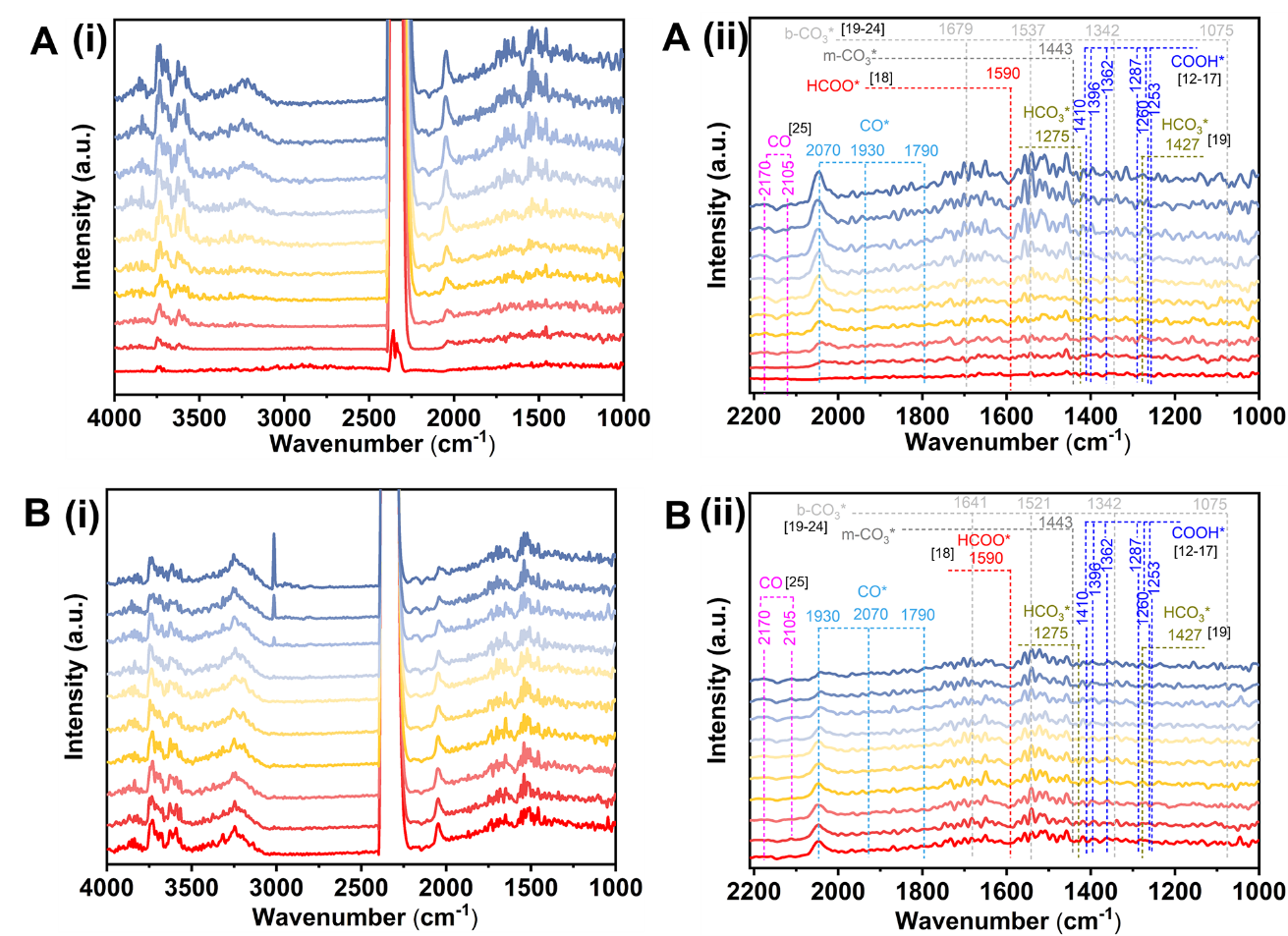


**Figure S14.** *In situ* DRIFTS full-spectrum and *in situ* DRIFTS spectrum from 1000 to 2200 cm^−1^ for Ni/NiSiO*_x_*(700) under (A) CO_2_/N_2_ and (B) subsequent injecting H_2_/N_2_ at 300 °C.


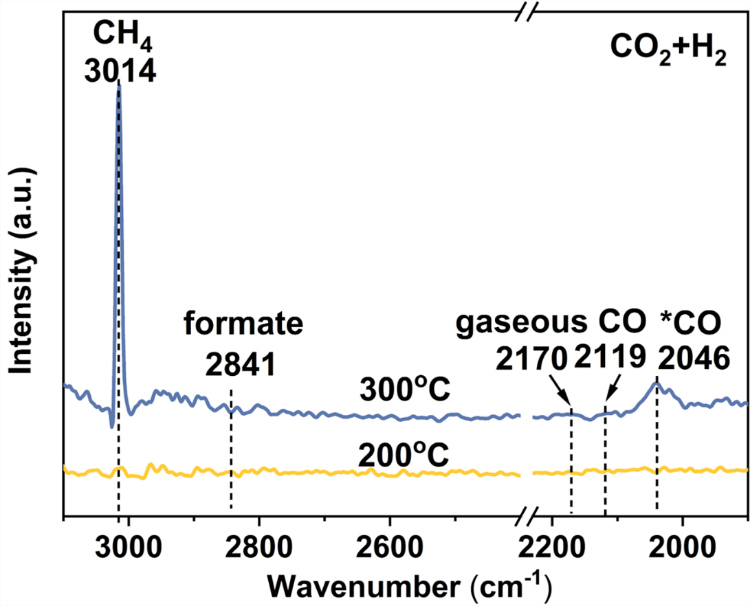


**Figure S15.** *In situ* DRIFTS spectra of Ni/NiSiO*_x_*(700) under MLT-RWGS reaction conditions (CO_2_ + H_2_).


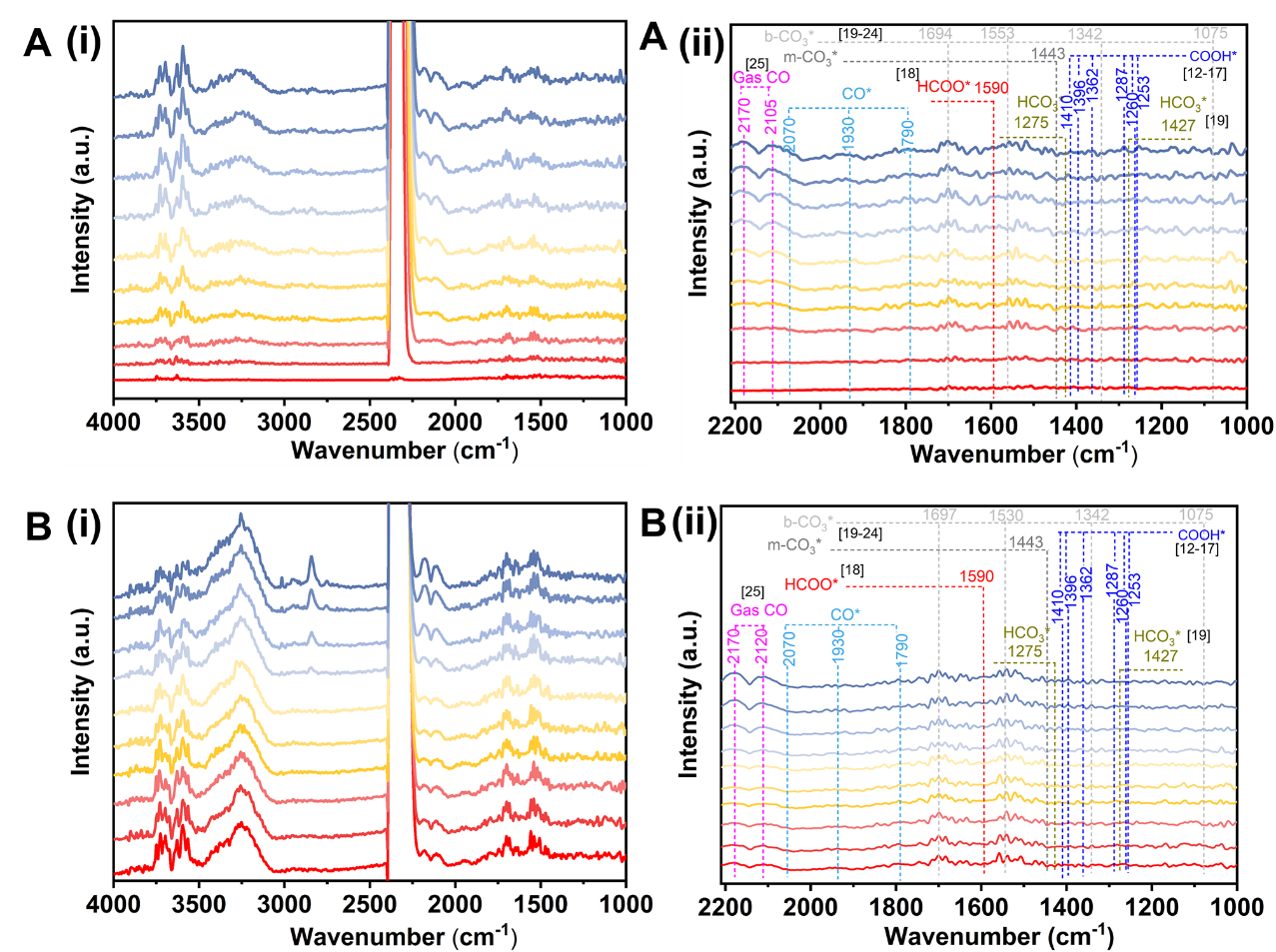


**Figure S16.** *In situ* DRIFTS full-spectrum and *in situ* DRIFTS spectrum from 1000 to 2200 cm^−1^ for a-Ni^δ+^-PSNS(400) under (A) CO_2_/N_2_ and (B) subsequent injecting H_2_/N_2_ at 300 °C


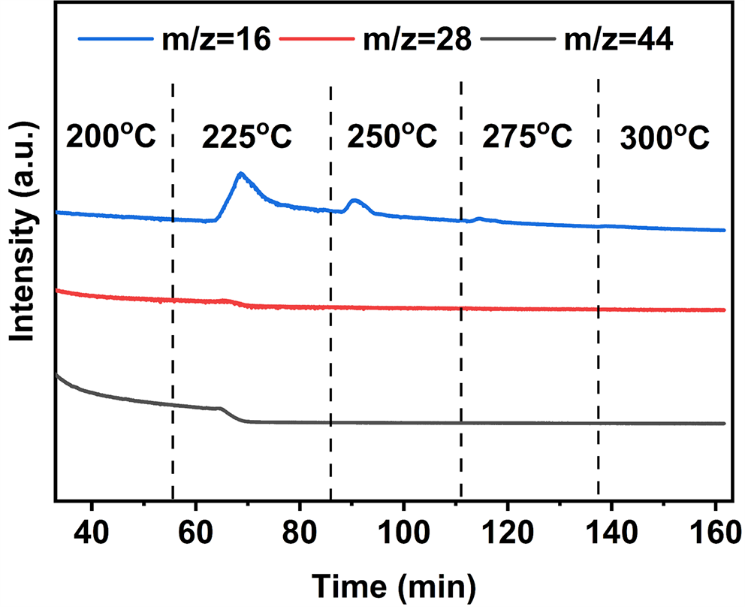


**Figure S17.** The formate conversion on a-Ni^δ+^-PSNS(400) simulated in a tube furnace coupled with a MASS spectrometer under programmed heating.


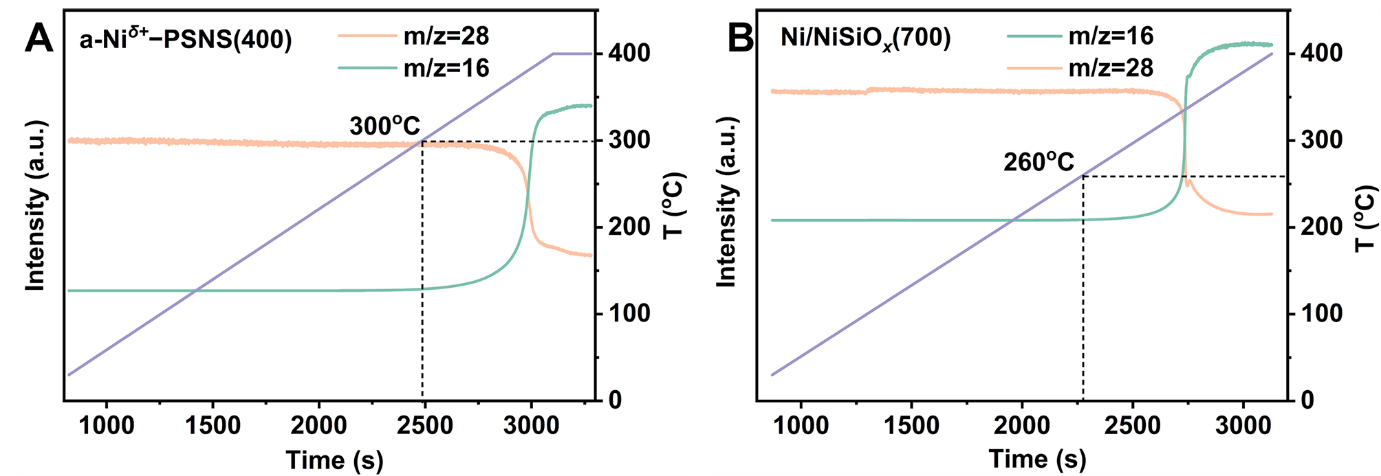


**Figure S18.**The temperature programmed surface reaction (TPSR) on (A) a-Ni*^δ+^*−PSNS(400) and (B) Ni/NiSiO*_x_*(700) under CO+H_2_ conditions.


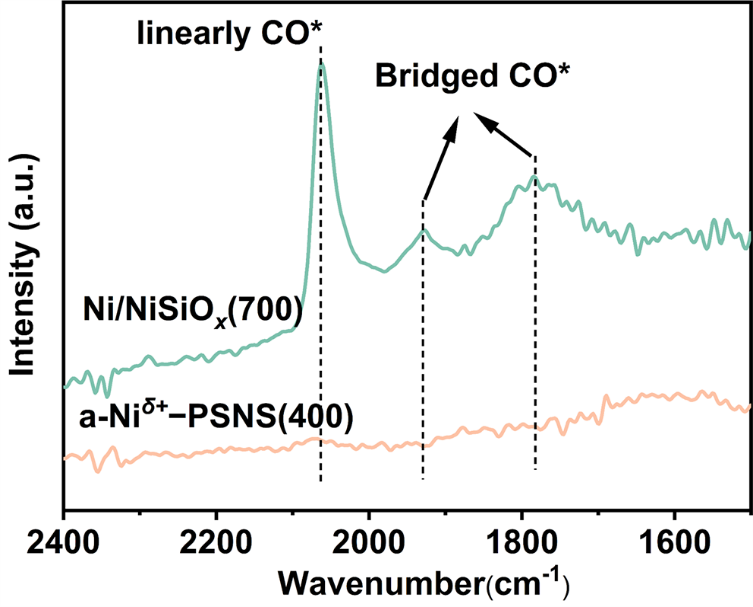


**Figure S19.** *In situ* DRIFTS recorded CO adsorption on a-Ni*^δ+^*−PSNS(400) and Ni/NiSiO*_x_*(700) at room temperature after flowing He for 30 min.


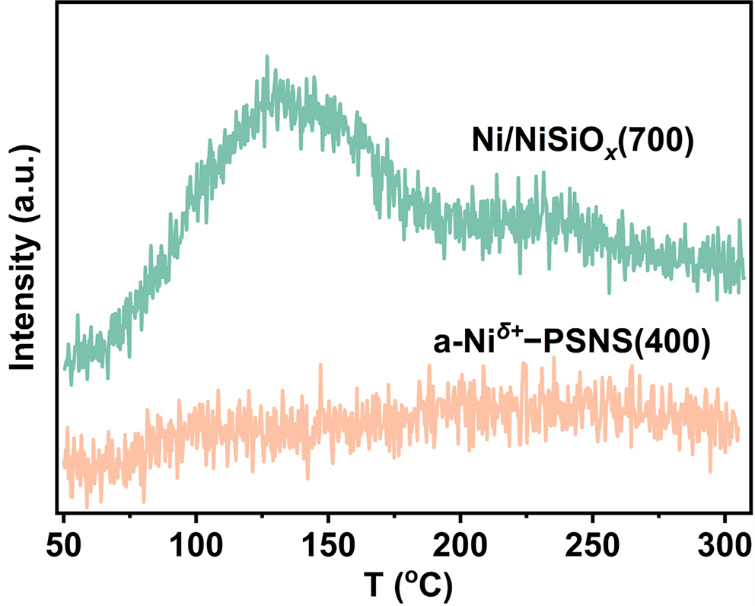


**Figure S20.** CO−TPD profiles of a-Ni*^δ+^*−PSNS(400) and Ni/NiSiO*_x_*(700).


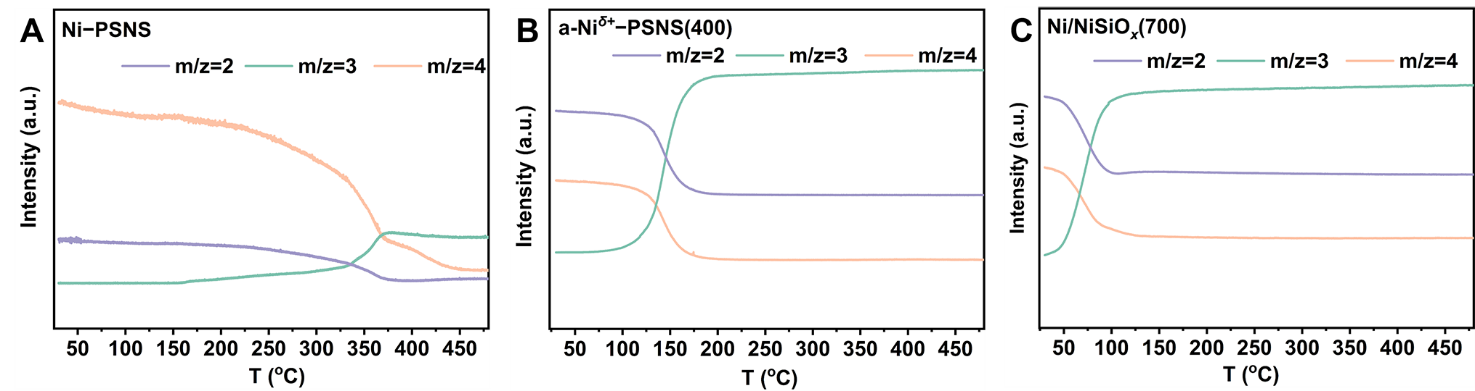


**Figure S21.** H−D exchange measurements of (A) Ni−PSNS, (B) a-Ni*^δ+^*−PSNS(400) and (B) Ni/NiSiO*_x_*(700).


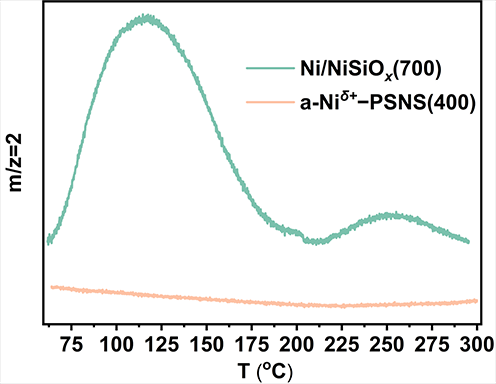


**Figure S22.** H_2_−TPD profiles of a-Ni*^δ+^*−PSNS(400) and Ni/NiSiO*_x_*(700).


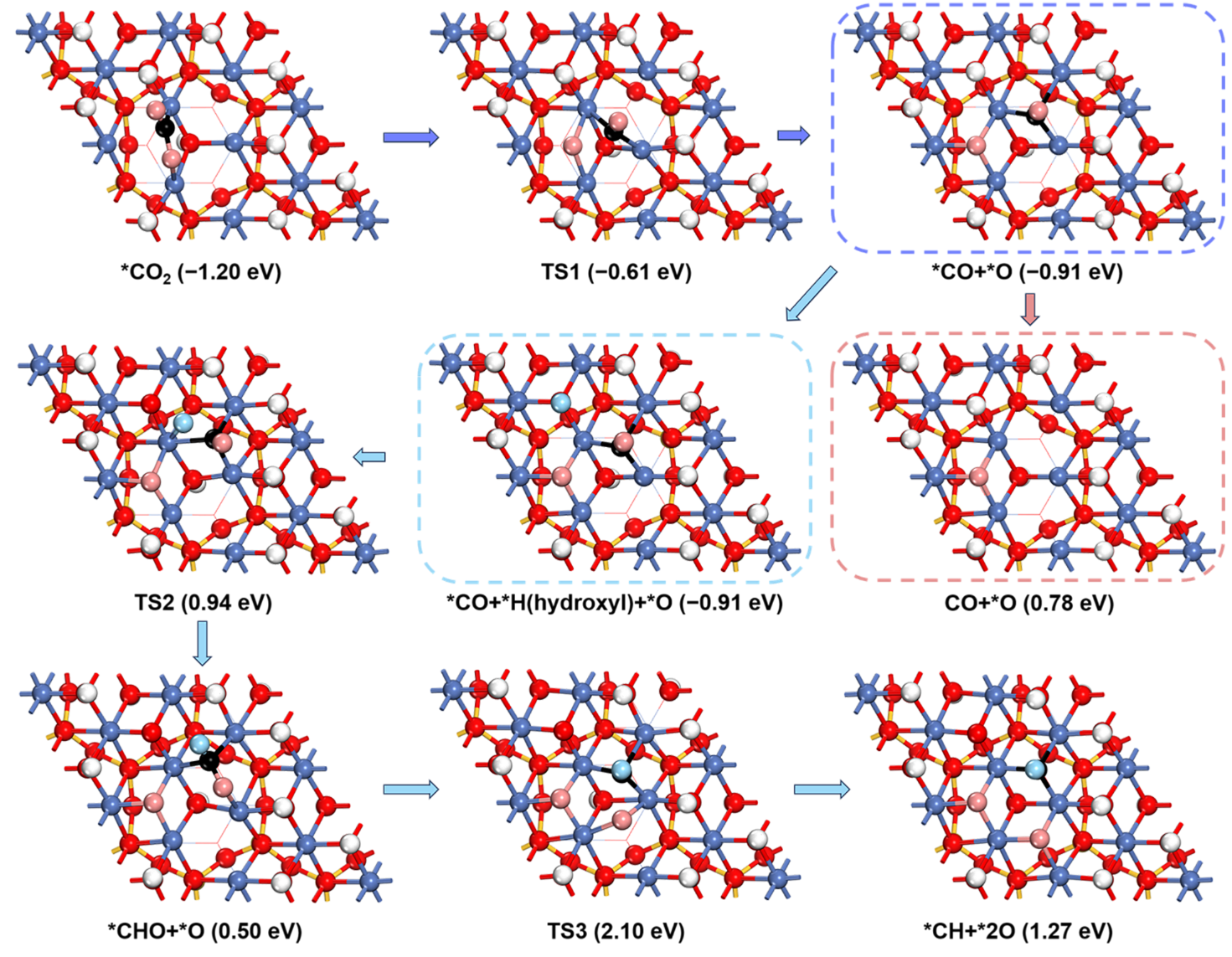


**Figure S23.** Geometric structures involved in the redox mechanism for CO_2_ hydrogenation on a-Ni*^δ+^*−PSNS(400) catalyst (O: red, Ni: purple, H of hydroxyl: white, C: black, H in adsorbate: blue, O in adsorbate: pink).


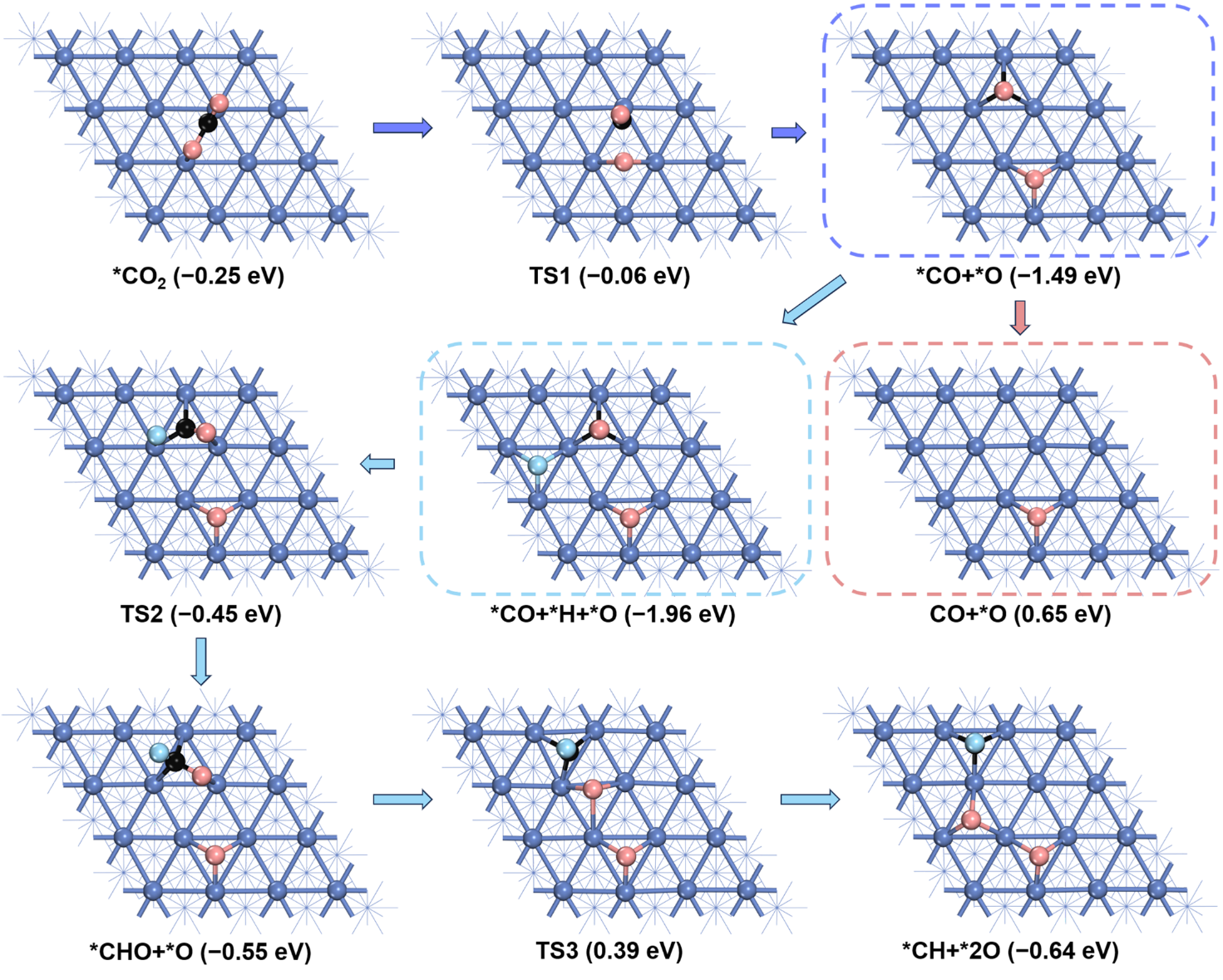


**Figure S24.** Geometric structures involved in the redox mechanism for CO_2_ hydrogenation on Ni/NiSiO*_x_*(700) catalyst (Ni: purple, H: blue, C: black, O in adsorbate: pink).


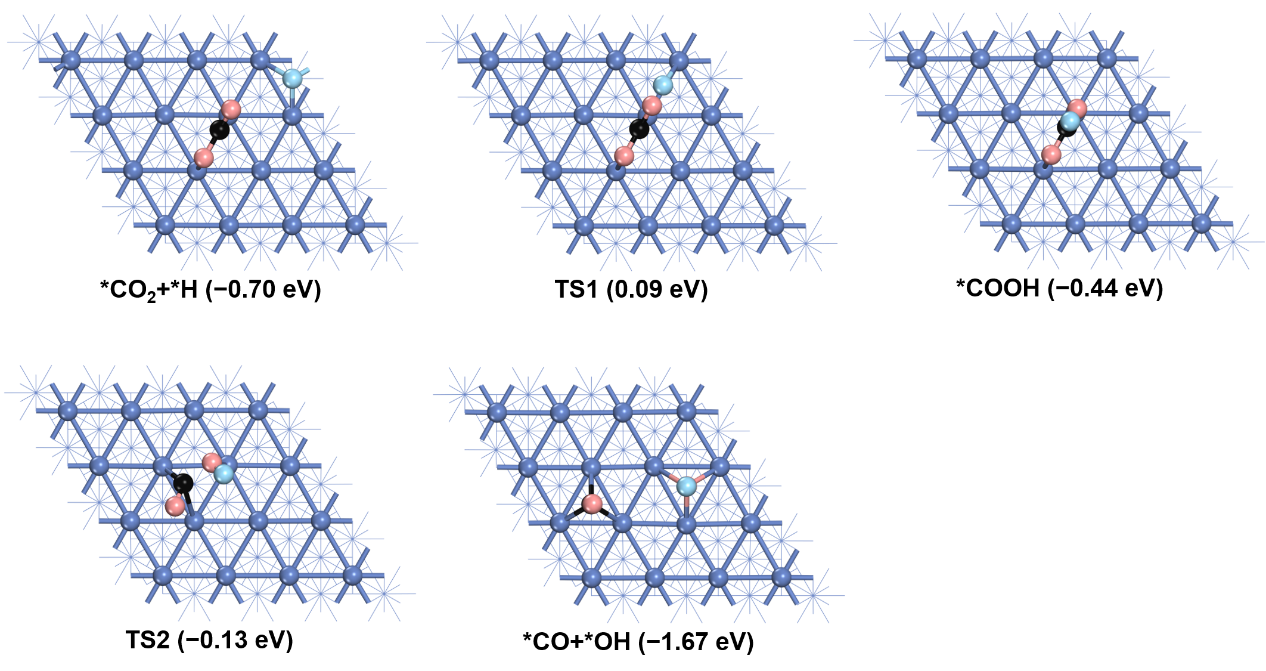


**Figure S25.** Geometric structures involved in H-assisted *COOH mechanism for CO_2_ hydrogenation on Ni/NiSiO*_x_*(700) catalyst (Ni: purple, H: blue, C: black, O in adsorbate: pink).


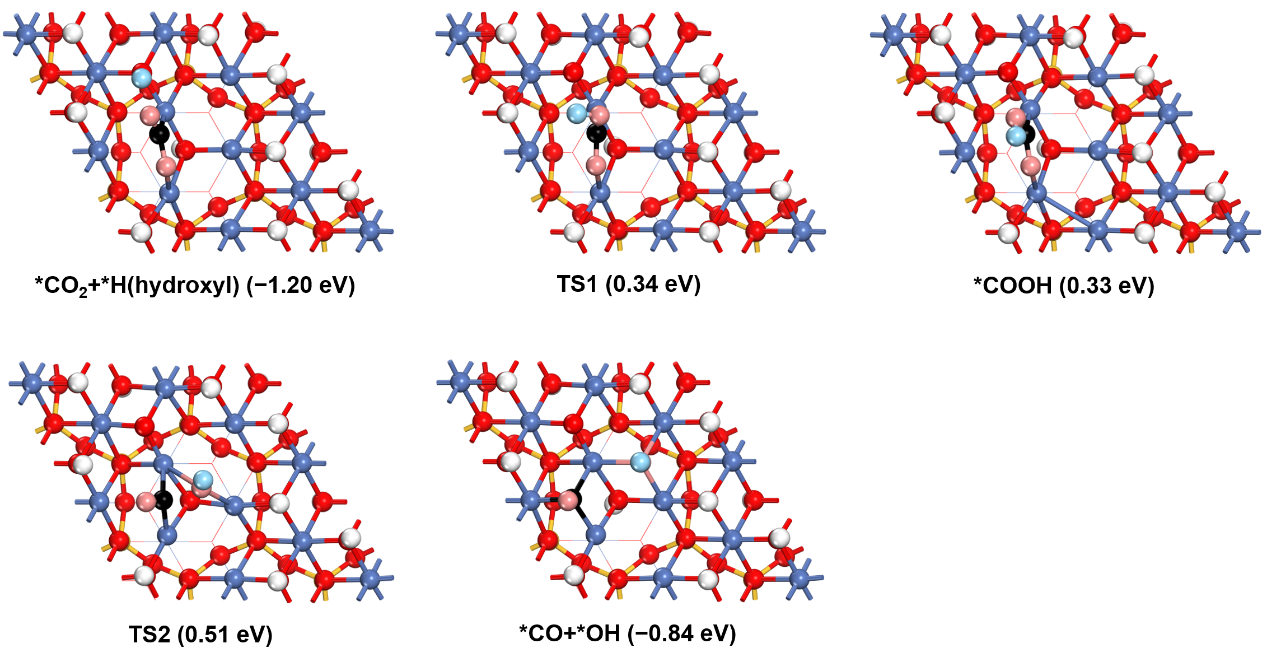


**Figure S26.** Geometric structures involved in H-assisted *COOH mechanism for CO_2_ hydrogenation on a-Ni*^δ+^*−PSNS(400) catalyst (Ni: purple, H: blue, C: black, O in adsorbate: pink).


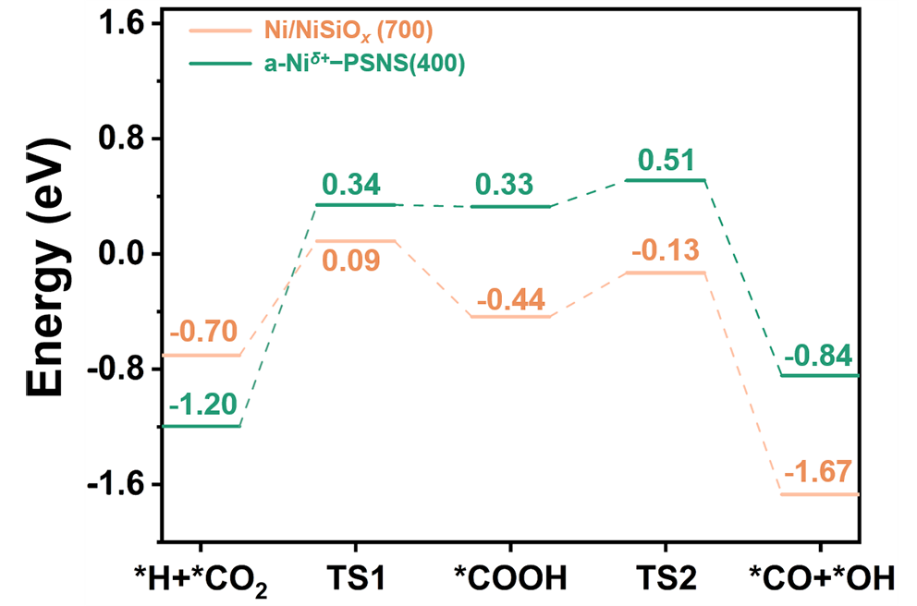


**Figure S27.** Potential energy diagrams of the CO_2_ hydrogenation into CO through H-assisted *COOH mechanism on the a-Ni*^δ+^*−PSNS(400) and Ni/NiSiO*_x_*(700) surfaces.


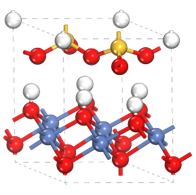


**Figure S28.** Crystal structure of Si_2_Ni_3_H_4_O_9_ (Si: orange, O: red, Ni: purple, H: white).


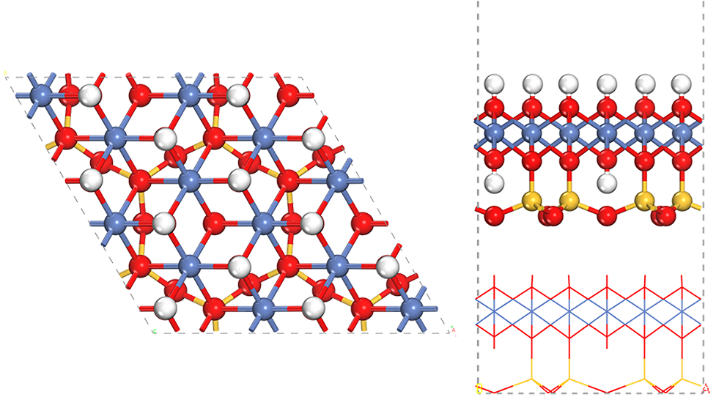


**Figure S29.** Top and side views of the (001) surface of Si_2_Ni_3_H_4_O_9_ (Si: orange, O: red, Ni: purple, H: white).


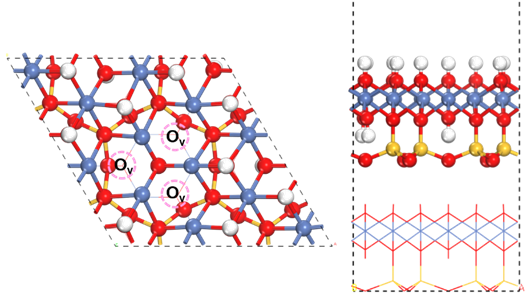


**Figure S30.** Top and side views of the (001) surface of Si_2_Ni_3_H_4_O_9_ after removing the outermost three hydroxyl groups (Si: orange, O: red, Ni: purple, H: white).


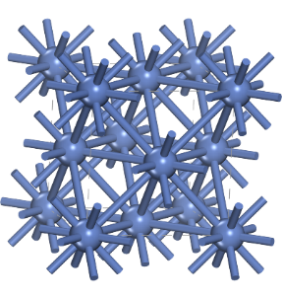


**Figure S31.** Crystal structure of Ni (Ni: purple).


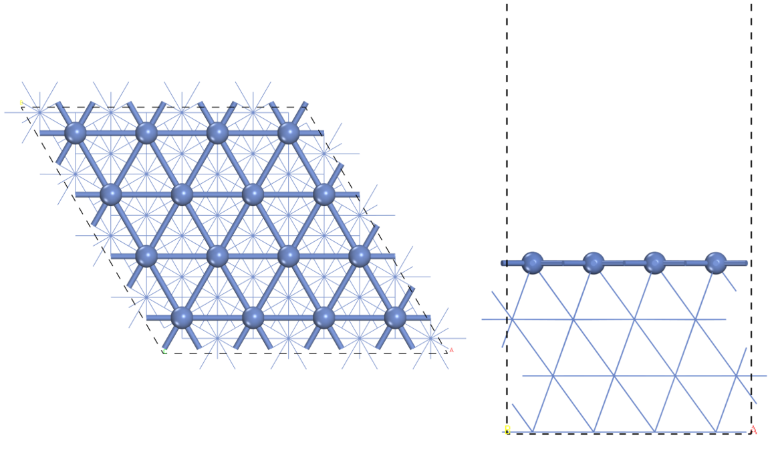


**Figure S32.** Top and side views of the Ni (111) surface (Ni: purple).

**Table S1.** Comparison of catalytic performance for the as-prepared and literature reported catalysts under MLT-RWGS reaction conditions (300−350 °C, and atmospheric pressure).

| catalysts | T  (°C) | P  (MPa) | H_2_/CO_2_ | WHSV  (mL g^-1^ h^-1^) | WHSV_CO2_  (mL g^-1^ h^-1^) | X_CO2_  (%) | S_CO_  (%) | STY  (mmol_CO_ g_cat_^-1^ h^-1^) | Ref. |
| --- | --- | --- | --- | --- | --- | --- | --- | --- | --- |
| a-Ni*^δ+^*−PSNS(400) | 300 | 0.1 | 1 | 10000 | 4500 | 11 | 92 | 21 | This work |
| a-Ni*^δ+^*−PSNS(400) | 300 | 0.1 | 1 | 300000 | 13500 | 4.3 | 95.7 | 184 | This work |
| Ni/MgAlO_x_-550 | 350 | 0.1 | 4 | 36000 | 6000 | 4.9 | 96.6 | 12.7 | [26] |
| Ni/ZnO | 300 | 0.1 | 4 | 36000 | 6000 | 1 | 99 | 3.2 | [27] |
| Ni/SiO_2_ | 350 | 0.1 | 1 | 120000 | 60000 | 10 | 40 | 107.1 | [28] |
| 20Ni20Zn/Al_2_O_3_ | 300 | 0.1 | 4 | 30000 | 4500 | 5 | 96 | 9.64 | [29] |
| Ni/γ-Mo_2_N | 300 | 0.1 | 3 | 300000 | 60000 | 2 | 100 | 53.6 | [30] |
| Ni/Ga_2_O_3_/Al_2_O_3_ | 350 | 0.1 | 4 | 30000 | 3000 | 7 | 97 | 9.1 | [31] |
| Ni/Ga_2_O_3_-rod | 350 | 0.1 | 4 | 30000 | 3000 | 3 | 99 | 4 | [31] |
| Ni/p-ZnO | 310 | 0.1 | 4 | 36000 | 7200 | 6.5 | 90.9 | 12.7 | [32] |
| Ni/Sm_2_O_3_ | 300 | 0.1 | 4 | 36000 | 6000 | 5 | 99 | 13.3 | [33] |
| Ni/ZrO_2_-80 | 300 | 0.1 | 4 | 13500 | 1500 | 4 | 100 | 2.7 | [34] |
| Ni-SAs/N-CNTs | 350 | 0.1 | 3 | 12000 | 3000 | — | 99.3 | 0.01 | [35] |
| Ni/Ce_0.9_Sn_0.1_O*_x_* | 300 | 0.1 | 4 | 36000 | 7200 | 5 | 98 | 15.8 | [36] |
| NiMgO*_x_* | 300 | 3 | 4 | 60000 | 10800 | — | 100 | 0.002 | [37] |
| Ni-P-4.2 | 300 | 0.1 | 4 | 30000 | 300 | 10 | 95 | 1.27 | [38] |
| Ni_A_WC | 300 | 0.1 | 3 | 12000 | 2400 | 1 | 100 | 1.07 | [39] |
| Ru_1_Ni_2.5_ | 300 | 0.1 | 4 | 37800 | 1890 | 1 | 100 | 0.84 | [40] |
| Ni-MAO-800-R | 300 | 0.1 | 4 | 90000 | 6000 | 5.2 | 94.8 | 13 | [41] |
| NiCu-Saponite | 300 | 0.1 | 4 | 15000 | 3000 | 1 | 100 | 2 | [42] |
| CsNi/CeO_2_ | 300 | 0.1 | 4 | 12000 | 2400 | 5 | 79 | 7.9 | [43] |
| NiP/CeAl | 300 | 0.1 | 4 | 12000 | 2400 | 10 | 12 | 2.4 | [44] |
| NiAu/SiO_2_ | 300 | 0.1 | 4 | 60000 | 14400 | 1 | 90 | 2.16 | [45] |
| 15CuCe | 300 | 0.1 | 3 | 400000 | 92000 | 2.5 | 100 | 102.7 | [46] |
| Cu/CeO_2_-hs | 300 | 0.1 | 3 | 300000 | 75000 | 1 | 100 | 33.5 | [47] |
| Cu/Ce_0.05_Mg_0.95_ | 300 | 0.1 | 3 | 300000 | 72000 | 2 | 100 | 64.3 | [48] |
| NiIn@MCM-41 | 300 | 1 | 4 | 30000 | 6000 | 2 | 97 | 5.2 | [49] |
| Cu-Al spinel | 300 | 0.1 | 5 | — | — | 4 | 100 | 4.14 | [50] |
| Cu10/CeO_2_ | 300 | 0.1 | 4 | 3000 | 600 | 6.8 | 100 | 1.82 | [51] |
| Ni-in-Cu | 300 | 0.1 | 3 | 40000 | 8000 | 4.7 | 100 | 16.92 | [52] |
| Cu-S-Ce | 300 | 0.1 | 3 | 48000 | — | 4 | 100 | 20.6 | [53] |
| 2Cu/AlOOH-900 | 300 | 0.1 | 3 | 36000 | — | 4 | 100 | 15.4 | [54] |
| 5%Co-N-C | 300 | 0.1 | 4 | 6000 | 1080 | 4 | 80 | 1.5 | [55] |
| Pt0.5W | 400 | 0.1 | 4 | 300000 | 30000 | 8.2 | 100 | 44.4 | [56] |
| Pd-Mn(22)/Al_2_O_3_ | 300 | 0.1 | 4 | 30000 | 3000 | 6 | 100 | 8.0 | [57] |
| Pt-Mo_2_N | 300 | 3 | 3 | 200000 | 48000 | 4 | 96 | 82.3 | [58] |
| Ru-MoO_3_ | 300 | 3 | 0.1 | 10000 | 300 | 15 | 95.1 | 1.91 | [59] |
| Pt-CeO_2_@SiO_2_ | 300 | 3 | 0.1 | 12000 | 2400 | 13.9 | 100 | 14.9 | [60] |
| Ru/a-TiO_2_ | 300 | 1 | 4 | 24000 | 4800 | 5.2 | 99 | 7.9 | [61] |
| Pt_1_/TiO_2_ | 250 | 0.1 | 4 | 80000 | 16000 | 2 | 100 | 3.6 | [62] |
| CoTe/TiO_2_ | 300 | 4 | 4 | 15000 | 3000 | 0.1 | 100 | 13.4 | [63] |
| Ir/TiO_2_-700 | 280 | 0.1 | 3.5 | 9000 | 2000 | N/A | 100 | 0.36 | [64] |

**Table S2.** Physicochemical properties of various Ni-based catalysts

| catalysts | BET surface area  (m^2^/g) | Ni species content*^a^* (wt %) | Mean Ni  particle size*^b^* (nm) | Metal Ni  dispersion*^c^* (%) | Surface metal Ni (or Ni*^δ+^*) concentration  (mmol/g) |
| --- | --- | --- | --- | --- | --- |
| Ni−PSNS | 146.5 | 5.4 | — | — | — |
| a-Ni^δ+^−PSNS(300) | 141.9 | 5.0 | — | — | 0.12 |
| a-Ni^δ+^−PSNS(400) | 123.1 | 5.0 | — | — | 0.25 |
| Ni/NiSiO*_x_*(500) | 126.5 | 5.1 | 1.8 | 33.2 | 0.16 |
| Ni/NiSiO*_x_*(600) | 123.9 | 5.1 | 2.9 | 19.0 | 0.16 |
| Ni/NiSiO*_x_*(700) | 107.8 | 5.1 | 5.7 | 14.5 | 0.12 |

^a^ Ni species content was determined by inductively coupled plasma−atomic emission spectroscopy (ICP−AES). ^b^ Mean Ni particle size of Ni/NiSiO*_x_*(500), Ni/NiSiO*_x_*(600), and Ni/NiSiO*_x_*(700) was determined by TEM images, while Ni particles were absent in Ni−PSNS, a-Ni*^δ+^*−PSNS(300), and a-Ni*^δ+^*−PSNS(400). ^c^ Metal Ni dispersion and surface metallic Ni concentration of Ni/NiSiO*_x_*(500), Ni/NiSiO*_x_*(600), and Ni/NiSiO*_x_*(700) were calculated based on the results of H_2_-pulse adsorption at room temperature (described in the Experimental Section); and dispersion of Ni*^δ+^* atoms in a-Ni*^δ+^*−PSNS(300) and a-Ni*^δ+^*−PSNS(400) cannot be obtained through H_2_-pulse adsorption due to their no obvious adsorption for hydrogen, while their surface Ni*^δ+^* atoms concentrations were obtained by calculating integral area ratio of Ni*^δ+^* fitting peak to Ni^2+^ fitting peak in *semi-in situ* Ni 2p_3/2_ XPS spectra combined with the Ni loading (described in the Experimental Section).

References

[1] G. Kresse, J. Furthmüller, Computational Materials Science 1996, 6, 15-50.

[2] G. Kresse, J. Furthmüller, Physical Review B 1996, 54, 11169-11186.

[3] P. E. Blöchl, Physical Review B 1994, 50, 17953-17979.

[4] G. Kresse, D. Joubert, Physical Review B 1999, 59, 1758-1775.

[5] J. P. Perdew, K. Burke, M. Ernzerhof, Physical Review Letters 1996, 77, 3865-3868.

[6] S. Grimme, J. Antony, S. Ehrlich, et al., J Chem Phys 2010, 132, 154104.

[7] G. Henkelman, B. P. Uberuaga, H. Jónsson, The Journal of Chemical Physics 2000, 113, 9901-9904.

[8] S. Zhang, H. Ma, L. Jia, et al., Applied Catalysis B: Environment and Energy 2025, 361.

[9] A. Wei, R. Zhang, Y. Qin, et al., The Journal of Physical Chemistry C 2022, 126, 18078-18089.

[10] V. Wang, N. Xu, J.-C. Liu, et al., Computer Physics Communications 2021, 267, 108033.

[11] C. Vogt, E. Groeneveld, B. M. Weckhuysen, Nat. Catal. 2018, 1, 127-134.

[12] J. K. Li, J. P. Dong, S. S. Liu, et al., Angew.Chem., Int. Ed. 2024, 63, e202412144

[13] X. Wan, Y. Li, Y. Chen, et al., Nat. Commun. 2024, 15, 1273.

[14] C. Lv, K. Huang, Y. Fan, et al., Nano Energy 2023, 111, 108384.

[15] X.-F. Qiu, H.-L. Zhu, J.-R. Huang, et al., J. Am. Chem. Soc. 2021, 143, 7242-7246.

[16] F. Ma, P. Zhang, X. Zheng, et al., Angew.Chem., Int. Ed. 2024, 63, e202412785.

[17] P. Zhao, H. Jiang, H. Shen, et al., Angew.Chem., Int. Ed. 2023, 62, e202314121

[18] C. X. Wang, H. X. Liu, C. J. Jia, Nat. Commun. 2024, 15, 8290.

[19] Z. Feng, C. Tang, P. Zhang, J. Am.Chem. Soc. 2023, 145, 12663.

[20] J. Wang, Liu, C. Li, Sci. Adv. 2017, 3, 1701290.

[21] C. Wu, X.-D. Wen, J. A. Rodriguez, D. Ma, Nat. Commun. 2020, 11, 5767.

[22] M.Wang, P.Wang, Sci. Adv. 2023, 9, adg0167.

[23] L. Fan, J. Zhang, K. Ma, J. Catal. 2021, 397, 116-127.

[24] Z. Zhang, L. Zhang, N. Yan, Mol. Catal. 2019, 475, 110461.

[25] F. C. Meunier, Cat Tod. 2023, 423,113863

[26] K. Feng, J. Tian, M. Guo, et al., Appl. Catal. B Environ. 2021, 292, 120191.

[27] S. Lin, Q. Wang, M. Li, et al., ACS Catal. 2022, 12, 3346-3356.

[28] H. C. Wu, Y. C. Chang, J. H. Wu, et al., Catal. Sci. Tech. 2015, 5, 4154-4163.

[29] L. Shen, W. Zhang, Y. Feng, et al., J. Mater. Chem. A, 2023, 11, 8248-8255.

[30] L. Lin, J. Liu, X. Liu, et al., Nat. Commun. 2021, 12, 6978.

[31] J. Gong, M. Chu, W. Guan, et al., Ind. Eng. Chem. Res. 2021, 60, 9448-9455.

[32] W. Liao, C. Tang, H. Zheng, et al., J. Catal. 2022, 407, 126-140.

[33] J. Zhao, X. Liu, D. Su, ACS Catal. 2024, 14, 3158-3168.

[34] Z. Zhang, Y. Zang, F. Gao, et al., New Journal of Chemistry 2022, 46, 22332-22340.

[35] D. Wang, Z. Yuan, X. Wu, et al., ACS Catal. 2023, 13, 7132-7138.

[36] Z. Zhang, Y. Tong, X. Fang, et al., Fuel 2022, 316.

[37] M. M. Millet, G. Algara Siller, E. Frei, J. Am. Chem. Soc. 2019, 141, 2451-2461.

[38] X. Wei, G. Johnson, Z. Wu, et al., J. Am. Chem. Soc. 2023, 145, 14298-14306.

[39] D. Ye, Z. Wu, T. Wang, et al., Adv. Mater. 2025, 2504431.

[40] S. Zou, Y. Liang, X. Zhang, et al., Angew.Chem., Int. Ed. 2024, 64, e202412835

[41] Z. Li, M. Xiao, X. Liu, et al., Small 2025, 21, 2411636.

[42] N. Nityashree, C. A. H. Price, L. Pastor-Perez, et al., Appl. Catal. B Environ. 2020, 261, 118241.

[43] J. Gandara-Loe, Q. Zhang, J. J. Villora-Picó, et al., Energy & Fuels 2022, 36, 6362-6373.

[44] Q. Zhang, L. Pastor-Pérez, J. J. Villora-Pico, et al., Fuel 2022, 323, 124301.

[45] X. Zhang, S. Han, B. Zhu, et al., Nat. Catal. 2020, 3, 411-417.

[46] H. X. Liu, S. Q. Li, C. J. Jia, Nat. Commun. 2022, 13, 867.

[47] Y. Zhang, L. Liang, Z. Chen, et al., Appl. Sur. Sci. 2020, 516, 146035.

[48] S. Li, X. Liu, J. Ma, et al., ACS Catal. 2025, 15, 3475-3486.

[49] S. Zhang, H. Ma, L. Jia, et al., Appl. Catal. B Environ. 2025, 361, 124646.

[50] Bahmanpour A M, Appl. Catal. B Environ. 2020, 266, 118669.

[51] C Zhang, P Li, Z Xiao, H Zhang, et al., ACS Sustainable Chem. Eng. 2022, 10, 4, 1524–1535.

[52] L X Wang, E Guan, Z Wang, et al., ACS Catal. 2020, 10, 16, 9261–9270.

[53] B Zhang, S Huang, Y Li, J Shen, et al., Appl. Catal. B Environ.2025, 366 ,125003.

[54] Yamei Fan, Rongtan Li, Beibei Wang, et al.,Nat Commun 2024,15,3046.

[55] Y Li, Z Zhao, W Lu, et al., Appl. Catal. B Environ.2023, 324,122298.

[56] W Bi, J Wang, R Zhang, Q Ge, et al., ACS Catal. 2024,14,11205−11217.

[57] S Naniwa, S Oka, S Iguchi, et al.,ACS Catal.2025,15,9257−9265.

[58] L Zhang, X Chu, L Liu, et al., Adv. Funct. Mater. 2025, 35, 2413043.

[59] H Xin, L Lin, R Li, et al., J. Am. Chem. Soc. 2022, 144, 11, 4874−4882

[60] J Lei, Z Wu, D Ye, et al., Angew. Chem. Int. Ed. 2025, 64, e202511913.

[61] X Li, J Lin, L Li, et al., Angew. Chem. Int. Ed. 2020, 59, 19983-19989.

[62] L Chen, RR Unocic, et al., AS Hoffman, JACS Au 2021, 1, 977-986.

[63] X Zhou, C Hansen, D Isler, et al., J. Am. Chem. Soc. 2025, 147, 26, 22309–22313.

[64] X Chen, X Su, et al., ACS Catal. 2017, 7, 4613-4620.
